# An evaluation of the recommendations for primary nutrition research addressing noncommunicable disease using the EPICOT+ framework: A cross‐sectional descriptive meta‐research study of Cochrane nutrition systematic reviews

**DOI:** 10.1002/cesm.12048

**Published:** 2024-03-18

**Authors:** Sheena Ruzive, Helene Theunissen, Solange Durão, Marianne E. Visser, Celeste E. Naude

**Affiliations:** ^1^ Division of Epidemiology and Biostatistics, Department of Global Health, Faculty of Medicine and Health Sciences Stellenbosch University Tygerberg South Africa; ^2^ Cochrane South Africa, South African Medical Research Council Tygerberg South Africa; ^3^ Centre for Evidence‐based Health Care, Division of Epidemiology and Biostatistics, Department of Global Health, Faculty of Medicine and Health Sciences Stellenbosch University Tygerberg South Africa

**Keywords:** Cochrane systematic reviews, EPICOT+ framework, noncommunicable disease, nutrition, research recommendations

## Abstract

**Background:**

Noncommunicable diseases (NCDs) are a global health problem and many risk factors associated with the development of NCDs are related to nutrition.

**Aims:**

This study aimed to describe and summarize the research recommendations for primary research made in Cochrane nutrition systematic reviews (SRs) addressing NCDs, according to the evidence, population, intervention, comparison, outcome, time stamp (date of search), study design, time frame (length of follow‐up) and burden of disease (EPICOT+) framework.

**Materials & Methods:**

We screened a database of Cochrane nutrition SRs (*n* = 692) in March 2021 to identify those SRs addressing the four main NCDs (cardiovascular diseases, cancer, chronic respiratory diseases, and diabetes) and their nutrition‐related risk factors (obesity, unhealthy diets). The “implications for research” sections of included SRs (and the “search methods” sections for the time stamp item) were analyzed using EPICOT+.

**Results:**

We included 150/692 SRs; most addressed cardiovascular diseases (38.6%), and cancer (17.3%). The EPICOT+ items with the most reported research recommendations were time stamp (98.7%), intervention (94.7%), study design (89.3%), and outcomes (86%), and the least reported were the time frame (52%), comparison (30%), and burden of disease (8%). Most SRs recommended more studies overall (98.7%), assessing specific interventions (93.3%) (e.g., specific foods/food groups and diets/dietary patterns, micronutrient/complementary supplements, nutrition education techniques, and policies/programs) and a range of clinical and patient‐related outcomes (84.7%). Recommendations related to study design and quality included the need for more randomized (72.7%), better quality (55.3%), larger (44.7%), and better reported (26%) studies with a long‐term duration of follow‐up (50.7%).

**Discussion:**

Our findings show that research recommendations reported in Cochrane nutrition SRs addressing the four major NCDs and their nutrition‐related risk factors largely followed the EPICOT+ framework. For example, items that could be improved upon were the comparison and time frame items.

**Conclusion:**

These recommendations could contribute more significantly to planning future primary studies addressing important evidence gaps and limitations in the current evidence base.

## Plain Language Summary

Noncommunicable diseases (NCDs), or chronic diseases, are a global health problem. NCDs include heart disease, cancer, chronic respiratory disease, and diabetes. Many of the factors associated with the development of NCDs are related to nutrition. In a database of Cochrane nutrition systematic reviews (SRs) (*n* = 692) we identified 150 SRs, focusing on four types of NCDs (cardiovascular diseases, e.g., heart attacks and stroke, cancer, chronic respiratory disease, e.g., asthma and diabetes), and their nutrition‐related risk factors (obesity, unhealthy diets). We analyzed certain sections of these SRs to see whether the authors included certain items from the EPICOT+ framework. This framework is used for creating or finding the best practice evidence‐based research. This includes reference to the evidence, population, intervention, comparison, outcome, date of search, study design, duration of follow‐up, and burden of disease in their recommendations and search methods. We found that almost all SRs reported the literature search date (98.7%), the intervention(s) that need to be examined (94.7%), the study design(s) that would work best (89.3%), and the outcome(s) that need to be measured (86%) in future studies. Fewer authors described how long study participants should be followed up for (52%), the intervention(s) against which the intervention of interest needs to be compared to in future studies (30%) and the burden of disease or condition being addressed (8%). We also analyzed what the authors recommended. Most SRs recommended more studies on whether the specific foods or food groups, diets or dietary patterns, micronutrient or complementary supplements, nutrition education techniques, and policies or programs work for a variety of clinical and patient‐relevant outcomes. Many authors recommended large, randomized studies, with better quality and reporting, and following study participants for long periods of time. Our findings show that research recommendations reported by authors of Cochrane nutrition SRs largely followed the EPICOT+ framework. For example, items that could be improved were the comparison and the time frame items. Our summary of their recommendations could help with planning of future studies by showing where important gaps in the current nutrition evidence are.

## 1 INTRODUCTION

Noncommunicable diseases (NCDs) accounted for 74% of all deaths globally in 2019. Global health estimates indicate that 17 million people die annually from a NCD before the age of 70 years, with 86% of these deaths occurring in low‐ and middle‐income countries (LMICs). The four major groups of NCDs that contribute to over 80% of premature NCD deaths are cardiovascular diseases (CVDs), followed by cancer, chronic respiratory diseases, and diabetes [1, 2].

To halt the global rise in NCDs the World Health Organization (WHO) adopted a global action plan in 2013, aimed at governments and stakeholders, for the prevention and treatment of NCDs [3]. In 2019, the World Health Assembly extended the plan and called for the development of an implementation roadmap to accelerate progress on preventing and controlling NCDs. The roadmap supports actions to achieve a set of nine global targets that would have the greatest impact towards prevention and management of NCDs [4]. Many of the risk factors associated with NCDs stem from unhealthy lifestyles and are modifiable, including unhealthy diets, tobacco use, physical inactivity, harmful use of alcohol, and psychological stress [5, 6]. Modification of such lifestyle factors may contribute to reaching global targets, including a one‐third reduction in mortality from cardiovascular diseases, cancer, diabetes, and chronic respiratory disease by 2030, a similar reduction in the prevalence of raised blood pressure, and to halt the rise in the prevalence of obesity and diabetes. Other targets include reductions in dietary intake of sodium or salt, harmful use of alcohol, and physical inactivity [4].

A vast body of observational and experimental evidence has shown that people with healthier diets have a lower risk of developing NCDs and associated morbidity and mortality [5, 6, 7, 8, 9, 10, 11, 12, 13] and policies, strategies and action plans to reduce unhealthy diets and/or promote healthy diets are integral to reducing premature mortality from NCDs. The WHO's Global Action Plan and implementation roadmap for the prevention and control of NCDs highlights the need to use strategies based on the latest scientific evidence and/or best practice, cost‐effectiveness, affordability, and public health principles, while ensuring that recommendations are culturally acceptable [3, 4].

The best available evidence is commonly considered to be from rigorously conducted and reported systematic reviews (SRs). Cochrane SRs are regarded as high‐quality sources of synthesized scientific evidence, as they follow robust and standardized methodology to find and collate all evidence that fits prespecified eligibility criteria to answer a specific research question [14, 15], summarizing the overall certainty and results of this evidence. While different methods are used to identify, prioritize, and display recommendations for research [16], examining recommendations identified and reported in SRs is one of the established approaches [17, 18].

To this end, all Cochrane SRs include a section on the implications of the SR's findings for research, which is aimed at providing detailed information about further research needs, as well as the desirable nature of this research [15]. Most Cochrane SRs identify and describe residual uncertainty and are a rich source of information for further research. Examining the “implications for research” sections of Cochrane SRs can thus help inform research recommendations for primary research and for evidence synthesis [19]. However, there is variability in how these sections are drafted by different Cochrane SR authors, and thus in how research recommendations are formulated and reported [20].

The evidence, population, comparison, outcome, time stamp, study design, time frame, and burden of disease (EPICOT+) framework was developed to help standardize the reporting of research recommendations under the “implications for research” section of SRs, aiming to ensure they are specific and explicit, and thus more useful [21]. According to this framework, research recommendations should be made in relation to eight items: the current state of evidence (E), the population(s) to be studied (P), the intervention(s) (I) to be examined, the comparison(s) (C), the outcome(s) (O) of interest, and time stamp (T). The “+” refers to the study design, time frame, and the burden of disease, which are additional items to be considered and described when formulating recommendations. More details about these items are provided in Box 1.

Box 1: **Items of the EPICOT+ framework [21]**

**Table:** 

**E**	Evidence	Description of the current state of the evidence
**P**	Population	Description of the characteristics of population(s) that need to be included in future studies (e.g., age, gender, comorbidities, and specific inclusion criteria)
**I**	Intervention	Description of the intervention(s) that need to be examined by future studies (e.g., type, dose, frequency, duration)
**C**	Comparison	Description of the comparison intervention(s) that need to be included by future studies (e.g., placebo, other drugs or no intervention)
**O**	Outcome	What outcomes need to measured or reported in future research and how they should be measured
**T**	Time stamp	Date of literature search
**+**	Study design Time frame Burden of Disease	The study design(s) that would best suit the proposed future research The length of follow‐up and duration of intervention needed in future studies Consideration of the burden of disease of the condition being addressed

Considering the global nutrition‐related NCD burden, and the potential of relevant Cochrane SRs to identify research recommendations and inform research priorities, we aimed to analyze the adequacy of the reporting of research recommendations for primary research made in the “implications for research” sections of Cochrane nutrition SRs addressing the four major groups of NCDs (cardiovascular diseases, cancer, chronic respiratory disease and diabetes) and their nutrition‐related risk factors (e.g., obesity and unhealthy diets), according to the EPICOT+ framework. Additionally, we sought to summarize the main research recommendations from SRs included in each NCD category in terms of the framework items. These recommendations could contribute to the planning of future primary studies by addressing important evidence gaps and limitations in the current evidence base for the prevention and control of NCDs.

## 2 MATERIALS AND METHODS

We conducted a cross‐sectional descriptive meta‐research study of published Cochrane nutrition SRs.

The source of SRs for our analysis was a database of Cochrane SRs on nutrition‐relevant topics (https://nutrition.cochrane.org/evidence) (referred to as Cochrane nutrition SRs). This database is compiled by regular screening of all active recordsin the Cochrane Database of Systematic Reviews (CDSR), to identify nutrition‐relevant SRs and protocols for SRs using prespecified definitions (Box 2). Up to March 2021, after screening a total of 8320 active records in the CDSR 692 completed Cochrane nutrition SRs were included in the database.

Box 2: **Definitions applied to the Cochrane Database of Systematic Reviews for screening and selection of Cochrane nutrition systematic reviews for the database**

Nutrition systematic reviews were defined as those that investigated the effects of:

(1) Diets and dietary patterns; foods; formulated, fortified, or enriched foods or nutritional products and nutrients and bioactive non‐nutrients naturally in foods delivered orally, generally, or parenterally;
(2) Nutrition education, promotion, counselling, and programmes; coordination of care or delivery of foods or nutrients; and
(3) Any policies, programmes, or systems that influence outcomes clearly distinguishable as nutrition‐related (nutrition‐specific and nutrition‐sensitive).

For this study, we screened the 692 Cochrane nutrition SRs included in the database at March 2021 to identify SRs addressing any of the four major groups of NCDs, namely cardiovascular diseases, cancer, chronic respiratory diseases, and diabetes or their nutrition‐related risk factors (e.g., obesity, unhealthy diets). These groupings were informed by the findings from the 2019 Global Burden of Disease Study [2]. Reviews were excluded if they included pregnant women as participants. If more than one version of a specific SR had been published, we included only the most recent version. Two researchers (SR and CEN/SD) independently screened the titles and abstracts in the database, and the full‐texts if necessary, to ascertain eligibility. Any disagreements were resolved by discussion and consensus among the researchers.

One researcher (SR) extracted data using a standardized and piloted data extraction form, and another reviewer (MV, SD, CN, and HT) checked the data extracted in 40% of the included SRs. Discrepancies were resolved through discussion. We extracted the following information from the included SRs: accession number, title, NCD grouping, NCD being addressed and/or nutrition‐related risk factor being addressed, and publication year. From the “implications for research” sections of the SRs, we extracted future research recommendations verbatim, and allocated each extracted description (sentences and phrases) to the most applicable item in the EPICOT+ framework, namely evidence, population, intervention, comparison, outcomes, study design, time frame and burden of disease (Box 1). The time stamp, or date of search, was extracted from the “search methods” section of the reviews.

### 2.1 Statistical analyses

We analyzed the data using Microsoft Excel and used descriptive statistics to explore and present the results in tables and charts and using narrative summaries. First, each included SR was categorized according to the main NCD grouping (i.e., cardiovascular diseases, cancer, chronic respiratory diseases, diabetes) or the NCD‐related nutritional risk factor it addressed (i.e., obesity or unhealthy diets). Second, we organized the extracted research recommendations by grouping similar descriptions of recommendations into broader descriptive categories created for each EPICOT+ framework item (Box 3). For example, all recommendations articulating the need for additional primary studies in a specific population were grouped together into a broad category called “more studies in specific population(s) required” under the “Population” item of the EPICOT+ framework. Lastly, after grouping the extracted research recommendations according to the broad descriptive categories, we summarized the specific research recommendations for primary research from each SR according to the key EPICOT+ Framework items, namely populations (e.g., gender, age, disease stage), interventions (e.g., specific intervention components, modality of intervention administration), comparisons (e.g., placebo, standard care or other treatment), outcomes (e.g., adverse events, efficacy, mortality), study design (e.g., RCTs, quality of study, adherence to guidelines) and time frame (duration of study, length of follow up).

Box 3: **Broad descriptive categories created per EPICOT+ framework item, and used to group similar research recommendations about primary research extracted from the “implications for research” sections of included Cochrane nutrition systematic reviews**

**Table:** 

**EPICOT+ Framework Items**	**Broad descriptive categories used to group similar research recommendations**
Evidence (E)	More studies required overall
Population (P)	More studies in specific population(s) required More studies required in a specific setting
Intervention (I)	More studies with specific intervention(s) required
Comparison (C)	More studies with specific comparison(s) required
Outcome (O)	More studies with specific outcome(s) required
Design of Study (+)	More randomized studies required Multicentre trials More non‐randomized intervention studies required More observational studies required Qualitative studies required Systematic reviews required Cost effective and/or economic evaluation studies required Factorial design required Interrupted time series required
Time frame Quality of Study	More studies with specific timeframes required (more long‐term/longer duration of follow‐up required) More studies with larger sample size/adequately powered studies More studies with better quality required More studies with better reporting

## 3 RESULTS

### 3.1 Characteristics of included SRs

**Figure 1:**
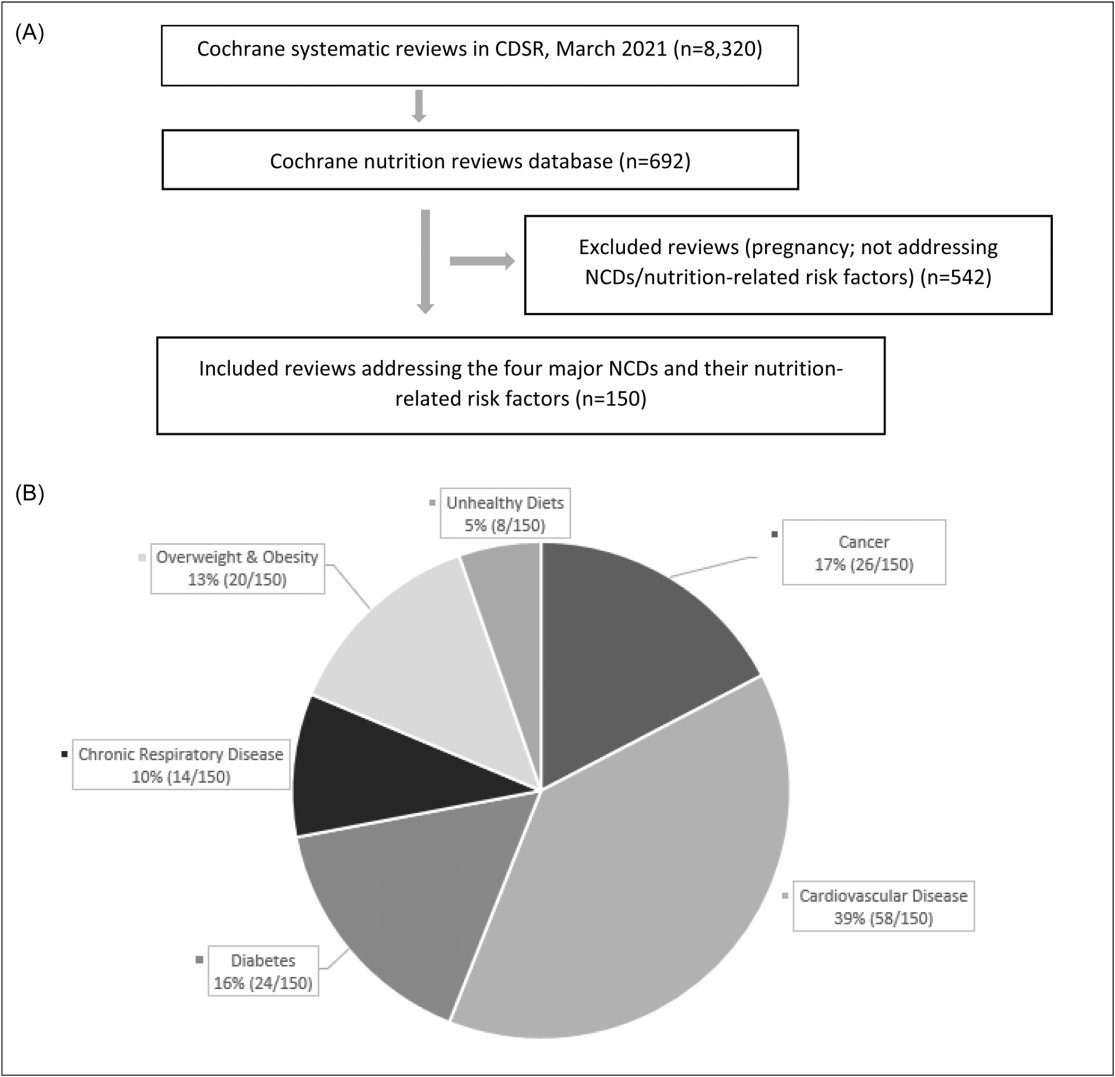
(A) Selection of eligible Cochrane nutrition systematic reviews (*n* = 150) and (B) their percentage distribution according to the four major noncommunicable disease (NCD) categories and their risk factors.

Of the 692 Cochrane nutrition SRs screened, 150 were eligible, including 122 that addressed the four major NCDs and 28 that addressed their associated nutrition‐related risk factors (Figure 1).

Most SRs addressed cardiovascular diseases (*n* = 58, 38.6%), followed by cancer (*n* = 26, 17.3%), diabetes (*n* = 24, 16%), and overweight and obesity (*n* = 20, 13.3%). Fewer SRs addressed chronic respiratory diseases (*n* = 14, 9.3%) and unhealthy diets (*n* = 8, 5.3%). Supporting Information S1: Table 1 summarizes the included SRs, the NCD or nutritional risk factor they addressed, as well as their digital object identifier (DOI) and date of publication.

Included SRs were published between 1998 and 2021, and included populations of healthy people, at‐risk populations and people with the NCD(s) of interest. Six SRs included only healthy people, while all others included people with NCDs or mixed populations (i.e., healthy and/or at risk and/or people with NCDs). Examples of at‐risk populations included people at high risk of gastrointestinal cancer in SRs addressing cancer, or adults with essential hypertension in those addressing CVD. Examples of populations with NCDs included children and young people with malignant disease for SRs addressing cancer, adults with existing cardiovascular disease or individuals with allergic asthma in SRs addressing cardiovascular disease and chronic respiratory disease, respectively.

The included SRs addressed the following types of interventions:

(1) *Diets and dietary patterns*, for example, low bacterial diets for cancer [22], Mediterranean diet for cardiovascular disease [23], weight loss interventions for chronic respiratory diseases [24], low glycemic index or low glycemic load diets for diabetes [25], and overweight and obesity [26];
(2) *Food groups*, for example, whole grain cereals or fruit and vegetables for cardiovascular diseases [27, 28], green tea for cancer [29], or overweight and obese adults [30];
(3) *Single foods*, for example, cocoa powder for cardiovascular disease [31], and sweet potato for diabetes [32];
(4) *Macronutrient supplements or complete nutrition formulas*, for example, omega‐6 fats for cardiovascular disease [33], nutritional supplements for chronic respiratory disease [34], and enteral or parenteral feeding in SRs addressing cancer [35, 36];
(5) *Micronutrient supplements*, for example, selenium for cardiovascular diseases [37], antioxidants or vitamin D for cancer [38, 39], Vitamin C for chronic respiratory diseases [40], and zinc for diabetes [41];
(6) *Complementary or alternative supplements*, for example, Coenzyme Q10 for cardiovascular diseases [42], probiotics for cancer [43], cinnamon for diabetes [44], and chitosan for obesity and overweight [45];
(7) *Nutrition education and counselling*, for example, individual patient education for managing type 2 diabetes [46], and dietary counselling interventions to address overweight or obesity in children and adolescents [47, 48, 49] and
(8) *Policies, strategies, and programs*, for example, food taxation policies (e.g., sugar‐sweetened beverages, high‐fat foods) for promoting healthy dietary behavior change [50, 51], and strategies to improve the implementation of healthy eating, physical activity, and obesity prevention policies, practices or programs in the childcare or school‐based setting [52, 53].

Just under a quarter (22.7%, 34/150) of reviews included interventions addressing diets and dietary patterns, followed by 19% (29/150) of reviews including interventions around nutrition education and counselling, and 17.3% (26/150) micronutrient supplements (Table 1).

**Table 1:** A summary of the types of interventions examined by Cochrane nutrition SRs for each NCD category.

	Cardiovascular disease (*n* = 58)	Cancer (*n* = 26)	Diabetes (*n* = 24)	Chronic respiratory disease (*n* = 14)	Overweight/obesity (*n* = 20)	Unhealthy diets (*n* = 8)
*Intervention type*						
Diets and dietary patterns	14	2	7	7	4	0
Food groups	2	1	0	0	1	0
Single foods	2	0	2	0	0	0
Macronutrient supplements	0	5	1	1	0	0
Micronutrient supplements	12	7	3	4	0	0
Complementary/alternative supplements	9	4	4	2	2	0
Nutrition education and counselling	9	5	7	0	7	1
Policies, strategies, and programs	1	0	0	0	2	6
Combinations of the above	9	2	0	0	4	1

Abbreviations: NCD, noncommunicable diseases; SR, systematic reviews.

### 3.2 Reporting of research recommendations in the “implications for research” sections of Cochrane nutrition SRs according to the EPICOT+ framework items

**Figure 2:**
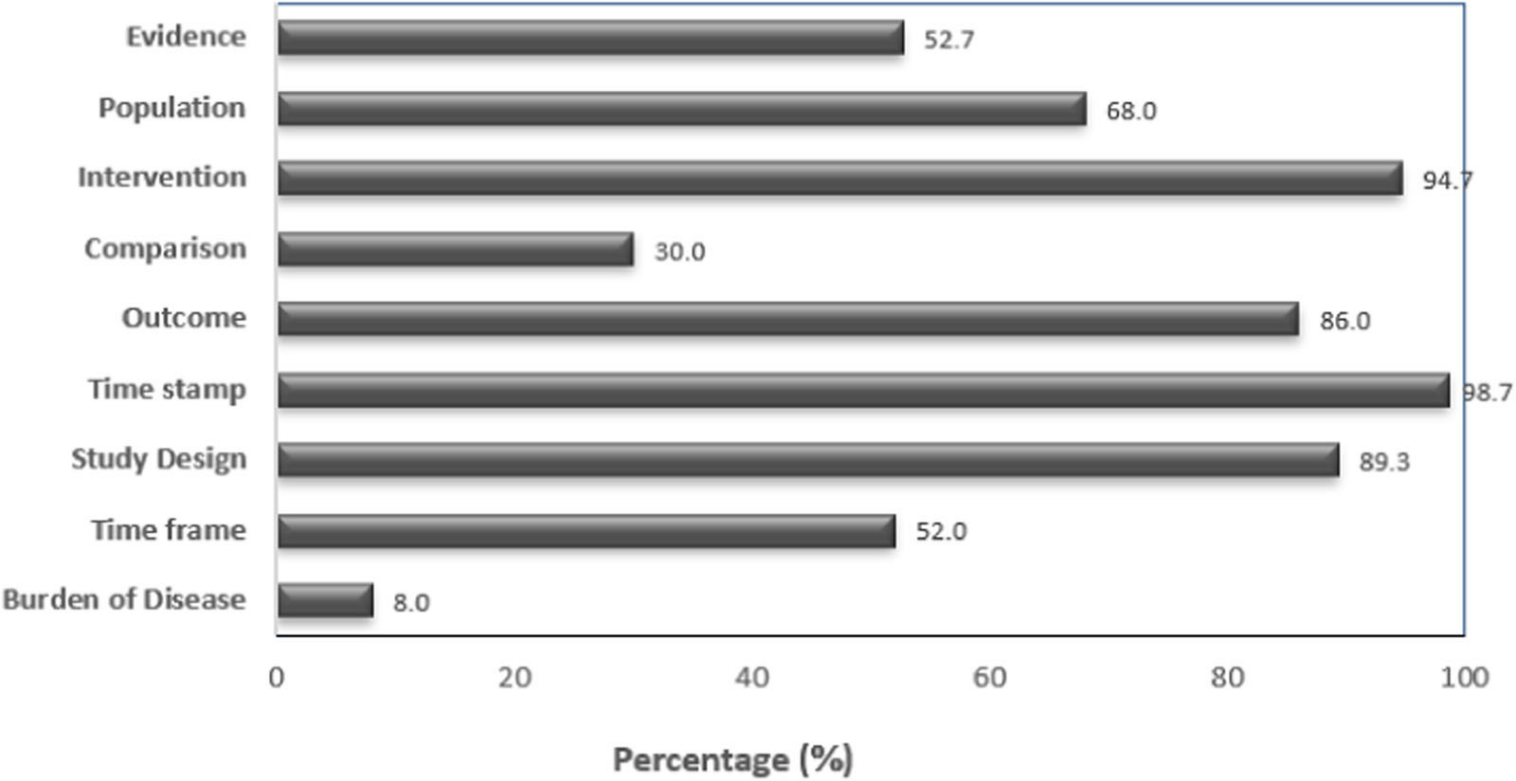
Proportion of Cochrane nutrition systematic reviews (*n* = 150) reporting research recommendations for each of the EPICOT+ framework items in their “implications for research” sections.

The EPICOT+ item with the most reported research recommendations across the SRs was the time stamp (*n* = 148, 98.7%) item, followed by the intervention (*n* = 142, 94.7%), study design (*n* = 134, 89.3%), and outcomes (*n* = 129, 86.0%) items (Figure 2). The EPICOT+ items with the least reported research recommendations were the time frame (*n* = 78, 52%), comparison (*n* = 45, 30.0%), and the burden of disease (*n* = 12, 8%) items. No included SR reported research recommendations with reference to all the EPICOT+ items.

### 3.3 Research recommendations for future primary research from the included Cochrane nutrition SRs according to descriptive categories (per EPICOT+ framework items)

The “implications for research” sections of all the included Cochrane nutrition SRs reported recommendations for future primary research. When grouped into broad descriptive categories, as per the EPICOT+ framework items (Box 3), most SRs highlighted the need for more studies overall (148/150, 98.7%) and for studies to address specific interventions of interest to the SR (140/150, 93.3%) (Figure 2). For example, in a SR of dietary saturated fat intake and cardiovascular disease, authors highlighted the need for specific dietary interventions to reduce or replace saturated fat [54]. Most SRs recommended that studies with more specific outcomes of interest to the SR were necessary (127/150, 84.7%) (e.g., adverse events), that more randomized studies were needed (109/150, 72.7%), and that more studies conducted in specific populations were needed (101/150, 67.3%) (e.g., in young populations). Approximately half of the SRs highlighted the need for more studies of better quality (83/150, 55.3%) and for more studies with specific timeframes (76/150, 50.7%). Nearly 50% of SRs called for studies with larger sample sizes (67/150, 44.7%), nearly a third for studies with comparisons specific to the SR of interest (44/150, 29.3%) (e.g., placebos), about a quarter for better reporting (39/150, 26.0%), and only about 20 for studies conducted in specific settings (23/150, 15.3%). In terms of study design, 31/150 (20.7%) SRs highlighted the need for more economic evaluation studies, but less than 10% of SRs highlighted the need for non‐randomized intervention studies (8/150, 5.3%), SRs (8/150, 5.3%), multicenter trials (7/150, 4.7%), observational studies (6/150, 4%), qualitative studies (2/150, 1.3%), studies with factorial design (2/150, 1.3%), or for interrupted time series studies (2/150, 1.3%).

### 3.4 Summary of research recommendations for future primary research from Cochrane nutrition SRs for each NCD category

A brief summary of research recommendations highlighted in the “Implications for research” sections of SRs for each NCD grouping and nutrition‐related risk factors is provided below. Please refer to supplementary tables for detailed summaries of specific research recommendations made by review authors for each NCD or risk factor category (Supporting Information S1: Tables S2‐S7).

#### 3.4.1 Cardiovascular disease (CVD)

Most (57/58, 98.3%) Cochrane nutrition SRs addressing CVD, highlighted the need for more studies, mainly for more randomized controlled trials (RCTs) (47/58, 81%) assessing the effectiveness of the interventions of interest (Figure 3A). More than half of the SRs (36/58, 62.1%) recommended more studies in specific populations, for example including individuals at high risk of CVD (e.g., populations with a low calcium intake [55]), those with CVD (e.g., hypertension [31, 56, 57, 58, 59, 60]) or from specific ethnic groups (e.g., Asian [61] or African [62] populations). Several SRs highlighted the need for research in both LMICs [33, 63, 64] and higher‐income countries [33, 65]. Almost all (57/58, 98.3%) reviews stated the need for more studies assessing specific interventions, such as those involving dietary patterns and advice (e.g., replacement of saturated fats with unsaturated dietary fats [54, 66], an increased intake of polyunsaturated fats, combined with a low trans‐fat intake [63]), food groups (e.g., dietary advice to increase fruit and vegetable consumption [27]) or single foods (e.g., fermented milk with higher bioavailability of the active peptides [67]). Recommendations also included the need for more studies investigating the supplementation of macronutrients (e.g., Alpha‐linolenic acid [65], omega‐3 polyunsaturated fatty acids, [68] creatine [69]), micronutrients (e.g., supplemental calcium of at least 1 g/day [55], magnesium and potassium [56, 70, 71], selenium [37]), or alternative supplements (e.g., isoflavones [61], Coenzyme Q10 [42, 72, 73]), and interventions at a policy or strategic level (e.g., regulatory reduction of salt and fat content of foods, improved availability of healthier foods [54, 66, 74]). More than three‐quarters (45/58, 77.6%) of the SRs, addressing a range of interventions, highlighted the need for studies to report specific outcomes, such as cardiovascular‐related mortality (13/58, 22.4%), outcomes and events (19/58, 32.8%), quality of life (11/58, 19%), and adverse events (10/58, 17.2%) (Supporting Information S1: Table S2).

Figure 3Proportion of Cochrane nutrition systematic reviews reporting research recommendations for each descriptive category created per EPICOT+ framework item (See Box 3) for (A) cardiovascular disease (*n* = 58), (B) cancer (*n* = 26), (C) diabetes (*n* = 24), (D) chronic respiratory disease (*n* = 14), (E) overweight & Obesity (*n* = 20), and (F) unhealthy diets (*n* = 8).

**Figure:**
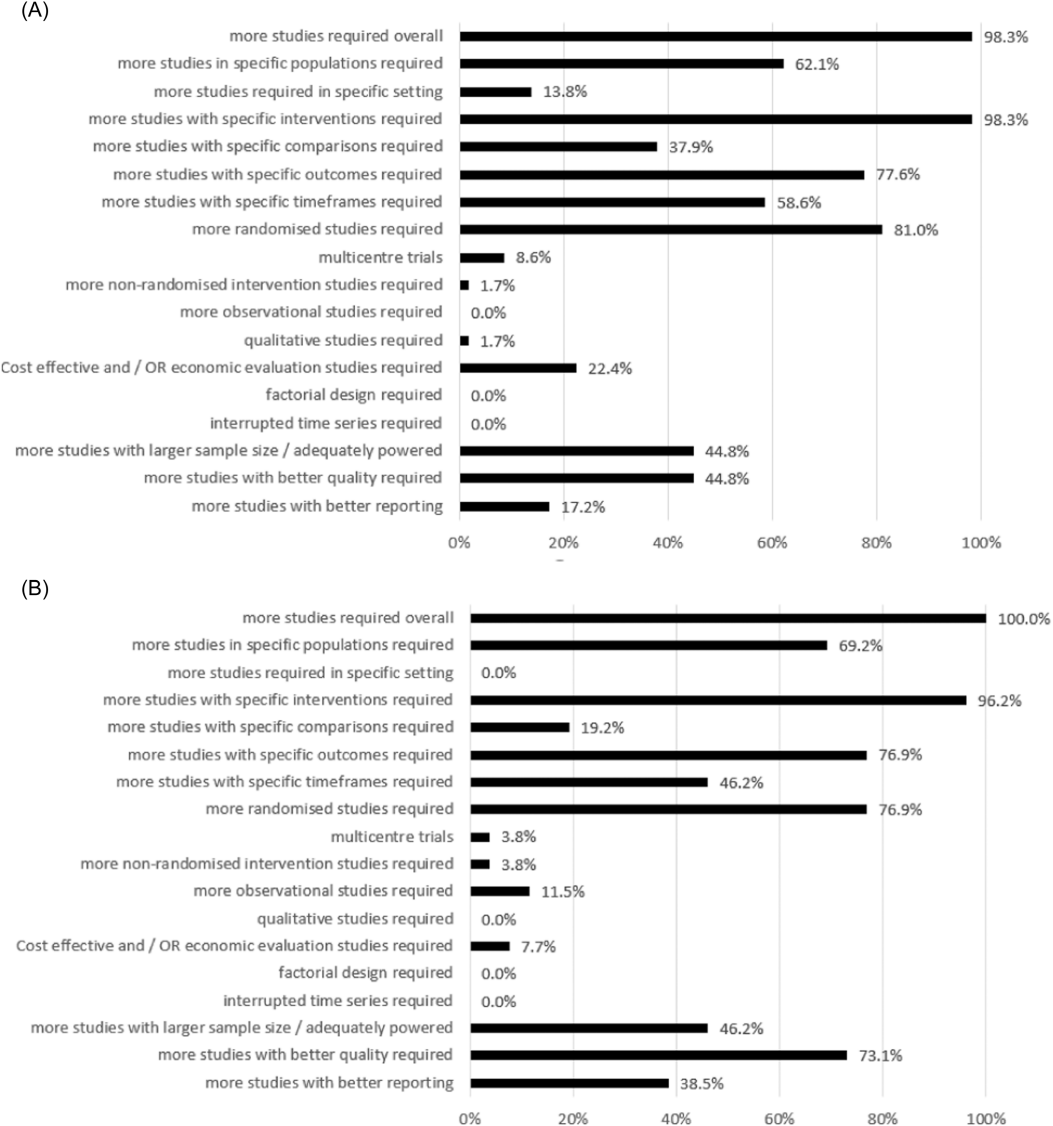

**Figure:**
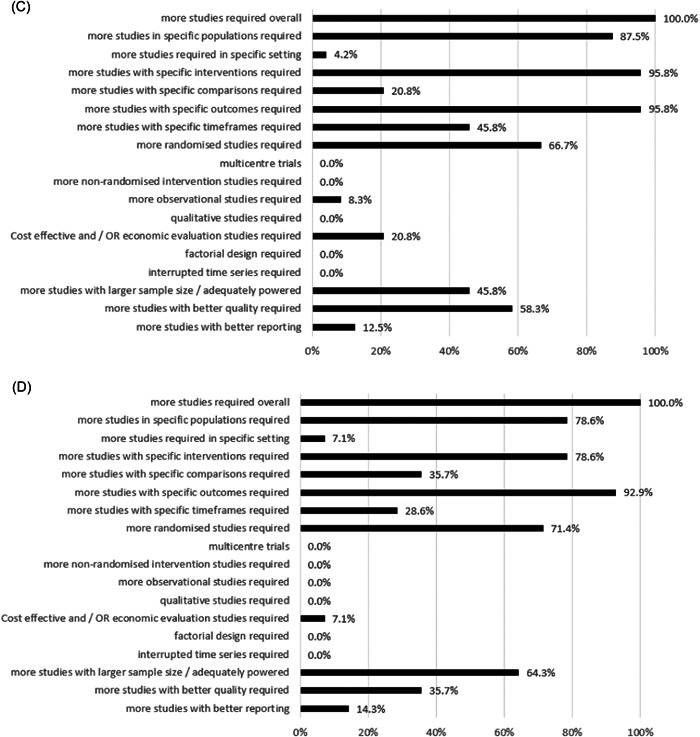

**Figure:**
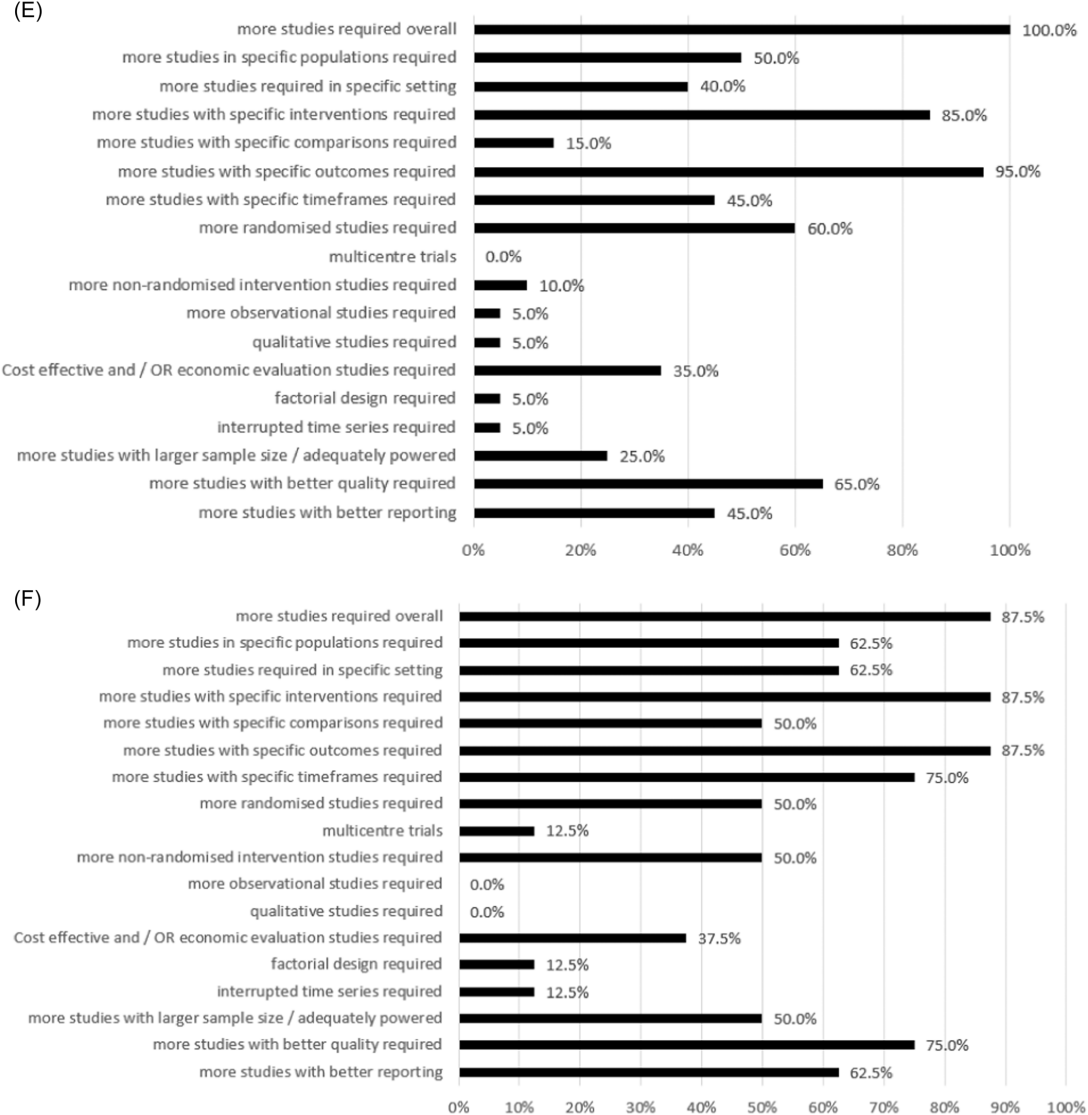

#### 3.4.2 Cancer

All 26 cancer‐related Cochrane nutrition SRs highlighted the need for more studies to be conducted overall, especially for RCTs (20/26, 76.9%) (Figure 3B). These SRs particularly raised the need for well‐designed, adequately powered RCTs. It was recommended that RCTs should adopt standard reporting guidelines, for example, “Standard Protocol Items: Recommendations for Interventional Trials” (SPIRIT) or “Consolidated Standards of Reporting Trials” (CONSORT) for the planning, execution, and reporting of these trials [75, 76]. Nearly three‐quarters of cancer‐related SRs (18/26, 69.2%) highlighted the need for studies to include specific populations; for example, to include homogenous study populations in terms of anticancer treatment and disease stage in studies assessing a variety of treatment interventions, including low bacterial diets, consumption of Reishi mushroom, supplementation of retinoic acid and parenteral nutrition [22, 36, 77, 78], as well as assessing the risk of cancer in specific groups, for example, in studies addressing vitamin D and selenium (e.g., young people, men, individuals with low vitamin D or selenium status) [39, 79]. Almost all SRs (25/26, 96.2%) highlighted the need for more studies assessing specific interventions; for example, those involving dietary patterns (e.g., intakes of dietary fiber of at least 30–40 mg/day [80]), the consumption of single foods (e.g., low to moderate doses of green tea [29]), the supplementation of macronutrients (e.g., the need for more palatable eicosapentaenoic acid formulations [81]), and the need to determine optimal dosages of micronutrients (e.g., selenium [82]), calcium [83], iron [84], or alternative supplements (e.g., flavonoids, probiotics [43, 85]). Most SRs highlighted the need for studies to be conducted that assess specific outcomes, for example, survival or mortality (9/26, 34.6%), quality of life (5/26, 19.2%), and safety or adverse events (6/26, 26.1%) (Supporting Information S1: Table S3).

#### 3.4.3 Diabetes

All diabetes‐related Cochrane nutrition SRs stated that more studies were required overall, particularly RCTs, studies of high quality and adequate power, and cost‐effectiveness studies (Figure 3C). Most SRs (21/24, 87.5%) indicated that studies in specific populations were required, for example, individuals with Type 2 Diabetes [32, 86, 87, 88, 89], those with diabetic kidney disease [90, 91], and children with diabetes [25, 44]. A total of 23/24 (95.8%) of SRs indicated that more studies assessing specific interventions of interest were required; examples of these included dietary patterns (e.g., modest sustained dietary salt reduction [91]), consumption of specific food groups (e.g., whole grains [92]), or foods (e.g., different varieties of sweet potato [32]). Recommendations also included the need for more studies investigating the optimal dosage of micronutrient supplements (e.g., vitamin B [90]), or exploring the effects of different species and preparations of cinnamon [44]. Similarly, 23/24 (95.8%) SRs called for more studies to report on specific outcomes, including clinical outcomes such as cardiovascular risk, insulin resistance, kidney function, neuropathy, and adverse events, as well as patient‐related outcomes, such as quality of life (10/24, 41.7%) and socioeconomic outcome measures (4/24, 16.7%) (Supporting Information S1: Table S4).

#### 3.4.4 Chronic respiratory disease

All 14 Cochrane nutrition SRs addressing chronic respiratory disease highlighted the need for more studies to be conducted (11/14, 78.6%) (Figure 3D), particularly in specific populations, for example, children and adolescents in LMICs [24], and adults with different types of asthma (e.g., chronic asthma [93], exercise‐induced asthma) [94, 95]. More than three quarters (11/14, 78.6%) of SRs highlighted the need for more studies assessing specific interventions including those aimed at achieving sustained weight loss [24], or the consumption of specific foods (e.g., an increased dietary intake of omega‐3 fatty acids by means of an increased consumption of fish [96]). Recommendations also included the need for further research to determine the optimal dosages of several vitamins (vitamins C, D, and E [34, 40, 95, 97]). Almost all (13/14, 92.9%) SRs raised the need for more studies reporting specific outcomes, such as exacerbation rates or symptoms of asthma (8/14, 57.1%), adverse effects (3/14, 21.4%), and quality of life (7/14, 50%) (Table S5).

#### 3.4.5 Overweight and obesity

All 20 Cochrane Nutrition SRs addressing overweight and obesity highlighted the need for more studies to be conducted, particularly in low‐ and middle‐income countries [98, 99, 100, 101, 102, 103], but also in high‐income settings [102] (Figure 3E). Half of the SRs (10/20, 50%) highlighted the need for more studies in specific populations, including obese and overweight children, adolescents [100], and adults [99, 104], as well as parents who are obese and their children [47]. Among 85% (17/20) of SRs stated that more studies assessing specific interventions were required, some of the recommended interventions included low‐fat diets and their long‐term safety [103], the application of the Transtheoretical model stages of change for achieving dietary change and sustained weight loss [104], or the use of e‐health systems and smartphones for weight management [99]. Most SRs (19/20, 95%) highlighted the need for more studies to assess specific outcomes, including obesity‐related comorbidities (4/20, 25%), quality of life (8/20, 40%), and adverse effects (4/20, 25%) (Supporting Information S1: Table S6).

#### 3.4.6 Unhealthy diets

Most SRs (7/8, 87.5%) (Figure 3F) highlighted the need for more studies overall to be conducted; 75% (6/8) of SRs recommended that studies in specific populations be conducted, for example, children from low‐income, minority or indigenous communities [105] or people from culturally diverse backgrounds [106, 107]. Most SRs (7/8, 87.5%) recommended that more studies assessing specific interventions be conducted. Examples of interventions included school‐based strategies involving behavioral interventions to improve health behavior [52], mass media interventions (e.g., to compare targeted mass media interventions with general mass media interventions) [106], and exposure to larger versus smaller‐sized portions, packages, individual units and tableware on selection and consumption of food [108]. Similarly, most SRs (7/8, 87.5%) raised the need for more studies assessing specific outcomes including adverse effects (e.g., increased family grocery costs [52, 105]) and behavior change [107, 109] (Supporting Information S1: Table S7).

## 4 DISCUSSION

This study assessed the reporting research recommendations according to the EPICOT+ framework items in the “Implications for research” sections of 150 Cochrane nutrition SRs that addressed the four major NCDs (cancer, cardiovascular diseases, chronic respiratory diseases, and diabetes) and nutrition‐related risk factors (obesity and unhealthy diets). In relation to the EPICOT+ items, almost all the included reviews reported the time stamp item of the EPICOT framework, as it is common practice for authors of Cochrane reviews to report the date of their literature search. We found that most research recommendations reported on interventions, study designs, and outcomes needed in future primary studies, and that the items least reported on were the time frame, comparison, and the burden of disease. These findings are consistent with those from a previous analysis of Cochrane HIV SRs, where the most reported EPICOT+ framework items were the population, intervention, outcome, and time frame, and the lesser reported items were the evidence, comparison, and burden of disease. According to Brown et al. [21] unlike the evidence, population, intervention, comparison, and outcome, reporting the burden of disease in the “implications for research” section is optional. This may be the reason fewer SRs report the burden of disease. However, considering the burden of disease when formulating research recommendations is important as this will inform researchers and policy makers of the settings for which urgent research is needed. Mbuagbaw et al. [20] hypothesized that the lesser reporting of the evidence item may be explained by the fact that authors allude to the evidence in the meta‐analyses, finding it repetitive to do the same in the “implications for research” section. In this respect, some of the included SRs in our analysis presented the results of their meta‐analyses as “Summary of Findings tables,” where the certainty of evidence for the main outcomes of each SR is evaluated by review authors using the Grading of Recommendations, Assessment, Development and Evaluations (GRADE) approach. This has become a prerequisite for conducting Cochrane SRs according to the Cochrane Handbook [110]. Even though there is no stated requirement that EPICOT+ items are reported in Cochrane SRs, this is a potentially useful approach that adds structure, specificity, and utility to the reporting of research recommendations.

From analyzing the “implications for research” section of these 150 Cochrane nutrition SRs, we found that most SRs highlight the need for more studies overall and the need for more randomized studies, particularly of high quality, adequately powered, and of longer duration. Most also highlighted the need for more studies assessing specific populations, interventions, and outcomes. These findings point out specific gaps and limitations in the existing primary evidence base for many of the questions addressed by the 150 Cochrane nutrition SRs.

The included SRs across all NCDs and nutrition‐related risk factors had research recommendations about methodological issues relating to the quality of studies, settings in which studies should be conducted, and the execution and reporting of studies. The SRs reported a need for future trials to minimize risk of bias by implementing adequate methods of randomization, allocation concealment, and blinding, intention‐to‐treat or per protocol analyses, and to explicitly describe these methods when reporting trials. The need for more research in LMICs was highlighted by SRs addressing cardiovascular diseases, chronic respiratory diseases, obesity, and overweight and unhealthy diets. Reviews addressing CVD and overweight and obesity also highlighted the need for more research in higher‐income countries. In terms of reporting, Cochrane nutrition SRs recommended that future primary studies use appropriate reporting guidelines, such as the SPIRIT guidelines for protocols for clinical trials [75], or the CONSORT guidelines for reporting clinical trials [76].

Our results are comparable with findings in a similar study in Cochrane SRs addressing HIV, which suggested the need for more RCTs overall, more studies assessing different outcomes and more studies assessing different interventions [20]. Very few SRs highlighted the need for future studies with a design other than randomized studies (i.e., observational, non‐randomized, and qualitative studies and SRs).

Addressing the recommendations highlighted in these Cochrane nutrition SRs in future primary studies addressing cancer, CVD, diabetes, chronic respiratory disease, overweight/obesity, and unhealthy diets will likely contribute to improving the quality and usefulness of this evidence base and prevent research waste.

Our study has some limitations. The search for nutrition SRs addressing the four major NCDs and their nutrition‐related risk factors was done up to March 2021 and thus we may have missed relevant Cochrane SRs published thereafter. The research recommendations we identified were restricted to those reported by Cochrane SR authors.

Our findings show that research recommendations reported in Cochrane nutrition SRs addressing the four major NCDs and their nutrition‐related risk factors largely followed the EPICOT+ framework. Across all NCD groupings, most SRs called for more studies, particularly randomized studies, and more studies assessing specific interventions and outcomes to help better answer the review questions. Research recommendations for primary research made in Cochrane SRs could potentially contribute to ensuring that future studies address important evidence gaps and limitations in current studies. This, in turn, would help improve the utility of future nutrition SRs to better serve the needs of healthcare decision‐makers.

## AUTHOR CONTRIBUTIONS

**Sheena Ruzive**: Data curation; formal analysis; methodology; writing—original draft; writing—review and editing. **Helene Theunissen**: Formal analysis; writing—review and editing. **Solange Durão**: Conceptualization; data curation; formal analysis; methodology; supervision; writing—review and editing. **Marianne E. Visser**: Data curation; formal analysis; methodology; writing—review and editing. **Celeste E. Naude**: Conceptualization; data curation; formal analysis; methodology; supervision; writing—review and editing.

## CONFLICT OF INTEREST STATEMENT

The authors declare no conflict of interest.

## PEER REVIEW

The peer review history for this article is available at https://www.webofscience.com/api/gateway/wos/peer-review/10.1002/cesm.12048.

## Supporting information

Supporting information.

## Data Availability

Data available on request from the authors.
