# Supplementary material for: An evaluation of the recommendations for primary nutrition research addressing noncommunicable disease using the EPICOT+ framework: A cross‐sectional descriptive meta‐research study of Cochrane nutrition systematic reviews
Source: Cochrane Evid Synth Methods. 2024 Mar 18;2(3):e12048. doi: 10.1002/cesm.12048 (PMC11795943; doi:10.1002/cesm.12048)
Supplement: Supplementary file 1 — Supporting information. [file CESM-2-e12048-s001.docx]

**Supplementary material**

Table S1: Overview of included Cochrane nutrition reviews

Table S2: Summary of research recommendations from Cochrane nutrition reviews addressing cardiovascular diseases

Table S3: Summary of research recommendations from Cochrane nutrition reviews addressing cancer

Table S4: Summary of research recommendations from Cochrane nutrition reviews addressing diabetes

Table S5: Summary of research recommendations from Cochrane nutrition reviews addressing chronic respiratory diseases

Table S6: Summary of research recommendations from Cochrane nutrition reviews addressing obesity and overweight

Table S7: Summary of research recommendations from Cochrane nutrition reviews addressing unhealthy diets

Supplementary reference list

| Table S1: Overview of included Cochrane nutrition reviews |  |  |  |
| --- | --- | --- | --- |
| **Cochrane review title** | **DOI** | **Year published** | **NCD or nutrition related risk factor** |
| **Cardiovascular Disease** | | | |
| Vitamin E for intermittent claudication | <https://doi.org/10.1002/14651858.CD000987> | 1998 | Cardiovascular disease |
| Dietary advice given by a dietitian versus other health professional or self-help resources to reduce blood cholesterol. | <https://doi.org/10.1002/14651858.CD001366> | 2003 | Cardiovascular disease |
| Advice to reduce dietary salt for prevention of cardiovascular disease. | <https://doi.org/10.1002/14651858.CD003656.pub2> | 2004 | Cardiovascular disease |
| Calcium supplementation for the management of primary hypertension in adults. | <https://doi.org/10.1002/14651858.CD004639.pub2> | 2006 | Cardiovascular disease |
| Magnesium supplementation for the management of primary hypertension in adults | <https://doi.org/10.1002/14651858.CD004640.pub2> | 2006 | Cardiovascular disease |
| Potassium supplementation for the management of primary hypertension in adults | <https://doi.org/10.1002/14651858.CD004641.pub2> | 2006 | Cardiovascular disease |
| Combined calcium, magnesium and potassium supplementation for the management of primary hypertension in adults | <https://doi.org/10.1002/14651858.CD004805.pub2> | 2006 | Cardiovascular disease |
| Weight reduction for primary prevention of stroke in adults with overweight or obesity | <https://doi.org/10.1002/14651858.CD006062.pub2> | 2006 | Cardiovascular disease |
| Lipid-lowering for peripheral arterial disease of the lower limb | <https://doi.org/10.1002/14651858.CD000123.pub2> | 2007 | Cardiovascular disease |
| Interventions in the management of serum lipids for preventing stroke recurrence | <https://doi.org/10.1002/14651858.CD002091.pub2> | 2009 | Cardiovascular disease |
| Low-fat diets for acquired hypercholesterolaemia | <https://doi.org/10.1002/14651858.CD007957.pub2> | 2011 | Cardiovascular disease |
| Creatine and creatine analogues in hypertension and cardiovascular disease | <https://doi.org/10.1002/14651858.CD005184.pub2> | 2011 | Cardiovascular disease |
| Multiple risk factor interventions for primary prevention of coronary heart disease | <https://doi.org/10.1002/14651858.CD001561.pub3> | 2011 | Cardiovascular disease |
| Fermented milk for hypertension | <https://doi.org/10.1002/14651858.CD008118.pub2> | 2012 | Cardiovascular disease |
| Garlic for the prevention of cardiovascular morbidity and mortality in hypertensive patients | <https://doi.org/10.1002/14651858.CD007653.pub2> | 2012 | Cardiovascular disease |
| Reduced or modiﬁed dietary fat for preventing cardiovascular disease | <https://doi.org/10.1002/14651858.CD002137.pub3> | 2012 | Cardiovascular disease |
| Isoflavones for hypercholesterolaemia in adults | <https://doi.org/10.1002/14651858.CD009518.pub2> | 2013 | Cardiovascular disease |
| Selenium supplementation for the primary prevention of cardiovascular disease | <https://doi.org/10.1002/14651858.CD009671.pub2> | 2013 | Cardiovascular disease |
| Increased consumption of fruit and vegetables for the primary prevention of cardiovascular diseases | <https://doi.org/10.1002/14651858.CD009874.pub2> | 2013 | Cardiovascular disease |
| Green and black tea for the primary prevention of cardiovascular disease | <https://doi.org/10.1002/14651858.CD009934.pub2> | 2013 | Cardiovascular Disease |
| Effect of longer-term modest salt reduction on blood pressure | <https://doi.org/10.1002/14651858.CD004937.pub2> | 2013 | Cardiovascular disease |
| Omega-3 fatty acids for intermittent claudication | <https://doi.org/10.1002/14651858.CD003833.pub4> | 2013 | Cardiovascular disease |
| Dietary advice for reducing cardiovascular risk | <https://doi.org/10.1002/14651858.CD002128.pub5> | 2013 | Cardiovascular disease |
| Garlic for peripheral arterial occlusive disease | <https://doi.org/10.1002/14651858.CD000095.pub2> | 2013 | Cardiovascular disease |
| Co-enzyme Q10 supplementation for the primary prevention of cardiovascular disease | <https://doi.org/10.1002/14651858.CD010405.pub2> | 2014 | Cardiovascular disease |
| Reduced dietary salt for the prevention of cardiovascular disease | <https://doi.org/10.1002/14651858.CD009217.pub3> | 2014 | Cardiovascular disease |
| Dietary interventions (plant sterols, stanols, omega-3 fatty acids, soy protein and dietary fibers) for familial hypercholesterolaemia | <https://doi.org/10.1002/14651858.CD001918.pub3> | 2014 | Cardiovascular disease |
| Oral zinc for arterial and venous leg ulcers | <https://doi.org/10.1002/14651858.CD001273.pub3> | 2014 | Cardiovascular disease |
| Multiple risk factor interventions for primary prevention of cardiovascular disease in low- and middle-income countries | <https://doi.org/10.1002/14651858.CD011163.pub2> | 2015 | Cardiovascular disease |
| Internet-based interventions for the secondary prevention of coronary heart disease | <https://doi.org/10.1002/14651858.CD009386.pub2> | 2015 | Cardiovascular disease |
| Nut consumption for the primary prevention of cardiovascular disease | <https://doi.org/10.1002/14651858.CD011583.pub2> | 2015 | Cardiovascular disease |
| Vitamin K for the primary prevention of cardiovascular disease | <https://doi.org/10.1002/14651858.CD011148.pub2> | 2015 | Cardiovascular disease |
| Self management programmes for quality of life in people with stroke | <https://doi.org/10.1002/14651858.CD010442.pub2> | 2016 | Cardiovascular disease |
| Blood pressure lowering efficacy of coenzyme Q10 for primary hypertension | <https://cochranelibrary.com/cdsr/doi/10.1002/14651858.CD007435.pub3/full> | 2016 | Cardiovascular disease |
| Population-level interventions in government jurisdictions for dietary sodium reduction | <https://cochranelibrary.com/cdsr/doi/10.1002/14651858.CD010166.pub2/full> | 2016 | Cardiovascular disease |
| Dietary fibre for the primary prevention of cardiovascular disease | <https://cochranelibrary.com/cdsr/doi/10.1002/14651858.CD011472.pub2/full> | 2016 | Cardiovascular disease |
| Vitamin C supplementation for the primary prevention of cardiovascular disease | <https://doi.org/10.1002/14651858.CD011114.pub2> | 2017 | Cardiovascular disease |
| Homocysteine-lowering interventions for preventing cardiovascular events | <https://doi.org/10.1002/14651858.CD006612.pub5> | 2017 | Cardiovascular disease |
| Effects of low sodium diet versus high sodium diet on blood pressure, renin, aldosterone, catecholamines, cholesterol, and triglyceride | <https://doi.org/10.1002/14651858.CD004022.pub5> | 2017 | Cardiovascular disease |
| Niacin for primary and secondary prevention of cardiovascular events | <https://cochranelibrary.com/cdsr/doi/10.1002/14651858.CD009744.pub2/full> | 2017 | Cardiovascular disease |
| Effect of cocoa on blood pressure | <https://doi.org/10.1002/14651858.CD008893.pub3> | 2017 | Cardiovascular disease |
| Whole grain cereals for the primary or secondary prevention of cardiovascular disease | <https://doi.org/10.1002/14651858.CD005051.pub3> | 2017 | Cardiovascular disease |
| Low glycaemic index diets for the prevention of cardiovascular disease | <https://doi.org/10.1002/14651858.CD004467.pub3> | 2017 | Cardiovascular disease |
| Polyunsaturated fatty acids for the primary and secondary prevention of cardiovascular disease | <https://doi.org/10.1002/14651858.CD012345.pub3> | 2018 | Cardiovascular disease |
| Omega 6 fatty acids for the primary and secondary prevention of cardiovascular disease | <https://doi.org/10.1002/14651858.CD011094.pub4> | 2018 | Cardiovascular disease |
| Swallowing therapy for dysphagia in acute and subacute stroke | <https://doi.org/10.1002/14651858.CD000323.pub3> | 2018 | Cardiovascular disease |
| Marine-derived n-3 fatty acids therapy for stroke | <https://doi.org/10.1002/14651858.CD012815.pub2> | 2019 | Cardiovascular disease |
| Mediterranean-style diet for the primary and secondary prevention of cardiovascular disease | <https://doi.org/10.1002/14651858.CD009825.pub3> | 2019 | Cardiovascular disease |
| mHealth education interventions in heart failure | <https://doi.org/10.1002/14651858.CD011845.pub2> | 2020 | Cardiovascular disease |
| Alcohol intake reduction for controlling hypertension | <https://doi.org/10.1002/14651858.CD010022.pub2> | 2020 | Cardiovascular disease |
| Effect of alcohol on blood pressure | <https://cochranelibrary.com/cdsr/doi/10.1002/14651858.CD012787.pub2/full> | 2020 | Cardiovascular disease |
| Reduction in saturated fat intake for cardiovascular disease | <https://doi.org/10.1002/14651858.CD011737.pub3> | 2020 | Cardiovascular disease |
| Omega-3 fatty acids for the primary and secondary prevention of cardiovascular disease | <https://doi.org/10.1002/14651858.CD003177.pub5> | 2020 | Cardiovascular disease |
| Vegan dietary pattern for the primary and secondary prevention of cardiovascular diseases. | <https://doi.org/10.1002/14651858.CD013501.pub2> | 2021 | Cardiovascular disease |
| Intermittent fasting for the prevention of cardiovascular disease | <https://doi.org/10.1002/14651858.CD013496.pub2> | 2021 | Cardiovascular disease |
| Long-term effects of weight-reducing diets in people with Hypertension | <https://doi.org/10.1002/14651858.CD008274.pub4> | 2021 | Cardiovascular disease |
| Coenzyme Q10 for heart failure | <https://doi.org/10.1002/14651858.CD008684.pub3> | 2021 | Cardiovascular disease |
| Calcium supplementation for prevention of primary hypertension | <https://doi.org/10.1002/14651858.CD010037.pub4> | 2022 | Cardiovascular disease |
| **Cancer** | | | |
| Selenium for alleviating the side effects of chemotherapy, radiotherapy and surgery in cancer patients | <https://doi.org/10.1002/14651858.CD005037.pub2> | 2006 | Cancer |
| Eicosapentaenoic acid (EPA, an omega-3 fatty acid from fish oils) for the treatment of cancer cachexia | <https://doi.org/10.1002/14651858.CD004597.pub2> | 2007 | Cancer |
| Dietary calcium supplementation for preventing colorectal cancer and adenomatous polyps | <https://doi.org/10.1002/14651858.CD003548.pub4> | 2008 | Cancer |
| Antioxidant supplements for preventing gastrointestinal cancers | <https://doi.org/10.1002/14651858.CD004183.pub3> | 2008 | Cancer |
| Lycopene for the prevention of prostate cancer | <https://doi.org/10.1002/14651858.CD008007.pub2> | 2011 | Cancer |
| Dietary flavonoid for preventing colorectal neoplasms | <https://doi.org/10.1002/14651858.CD009350.pub2> | 2012 | Cancer |
| Enteral feeding methods for nutritional management in patients with head and neck cancers being treated with radiotherapy and/or chemotherapy | <https://doi.org/10.1002/14651858.CD007904.pub3> | 2013 | Cancer |
| Vitamin D supplementation for prevention of cancer in adults | <https://doi.org/10.1002/14651858.CD007469.pub2> | 2014 | Cancer |
| Nutritional support in children and young people with cancer undergoing chemotherapy | <https://doi.org/10.1002/14651858.CD003298.pub3> | 2015 | Cancer |
| The role of iron in the management of chemotherapy-induced anemia in cancer patients receiving erythropoiesis-stimulating agents | <https://doi.org/10.1002/14651858.CD009624.pub2> | 2016 | Cancer |
| Educational interventions for the management of cancer-related fatigue in adults | <https://doi.org/10.1002/14651858.CD008144.pub2> | 2016 | Cancer |
| Nutritional interventions for survivors of childhood cancer | <https://doi.org/10.1002/14651858.CD009678.pub2> | 2016 | Cancer |
| Ganoderma lucidum (Reishi mushroom) for cancer treatment | <https://doi.org/10.1002/14651858.CD007731.pub3> | 2016 | Cancer |
| Low bacterial diet versus control diet to prevent infection in cancer patients treated with chemotherapy causing episodes of neutropenia | <https://doi.org/10.1002/14651858.CD006247.pub3> | 2016 | Cancer |
| Retinoic acid post consolidation therapy for high-risk neuroblastoma patients treated with autologous hematopoietic stem cell transplantation | <https://doi.org/10.1002/14651858.CD010685.pub3> | 2017 | Cancer |
| Dietary fibre for the prevention of recurrent colorectal adenomas and carcinomas | <https://doi.org/10.1002/14651858.CD003430.pub2> | 2017 | Cancer |
| Immunonutrition for patients undergoing surgery for head and neck cancer | <https://doi.org/10.1002/14651858.CD010954.pub2> | 2018 | Cancer |
| Interventions for weight reduction in obesity to improve survival in women with endometrial cancer | <https://doi.org/10.1002/14651858.CD012513.pub2> | 2018 | Cancer |
| Interventions to reduce acute and late adverse gastrointestinal effects of pelvic radiotherapy for primary pelvic cancers | <https://doi.org/10.1002/14651858.CD012529.pub2> | 2018 | Cancer |
| Home parenteral nutrition for people with inoperable malignant bowel obstruction | <https://doi.org/10.1002/14651858.CD012812.pub2> | 2018 | Cancer |
| Probiotics for the prevention or treatment of chemotherapy- or radiotherapy-related diarrhoea in people with cancer | <https://doi.org/10.1002/14651858.CD008831.pub3> | 2018 | Cancer |
| Selenium for preventing cancer | <https://doi.org/10.1002/14651858.CD005195.pub4> | 2018 | Cancer |
| Dietary interventions for adult cancer survivors | <https://cochranelibrary.com/cdsr/doi/10.1002/14651858.CD011287.pub2/full> | 2019 | Cancer |
| Perioperative nutrition for the treatment of bladder cancer by radical cystectomy | <https://doi.org/10.1002/14651858.CD010127.pub2> | 2019 | Cancer |
| Body weight management in overweight and obese breast cancer survivors | <https://doi.org/10.1002/14651858.CD012110.pub2> | 2020 | Cancer |
| Green tea (Camellia sinensis) for the prevention of cancer | <https://doi.org/10.1002/14651858.CD005004.pub3> | 2020 | Cancer |
| **Diabetes** | | | |
| Long-term non-pharmacological weight loss interventions for adults with type 2 diabetes mellitus | <https://doi.org/10.1002/14651858.CD004095.pub2> | 2005 | Diabetes |
| Protein restriction for diabetic renal disease | <https://doi.org/10.1002/14651858.CD002181.pub2> | 2007 | Diabetes |
| Dietary advice for treatment of type 2 diabetes mellitus in adults | <https://doi.org/10.1002/14651858.CD004097.pub4> | 2007 | Diabetes |
| Vitamin C and superoxide dismutase (SOD) for diabetic retinopathy | <https://doi.org/10.1002/14651858.CD006695.pub2> | 2008 | Diabetes |
| Whole grain foods for the prevention of type 2 diabetes mellitus | <https://doi.org/10.1002/14651858.CD006061.pub2> | 2008 | Diabetes |
| Omega-3 polyunsaturated fatty acids (PUFA) for type 2 diabetes mellitus | <https://doi.org/10.1002/14651858.CD003205.pub2> | 2008 | Diabetes |
| Low glycaemic index, or low glycaemic load, diets for diabetes mellitus | <https://doi.org/10.1002/14651858.CD006296.pub2> | 2009 | Diabetes |
| Individual patient education for people with type 2 diabetes mellitus | <https://doi.org/10.1002/14651858.CD005268.pub2> | 2009 | Diabetes |
| Altered dietary salt intake for preventing and treating diabetic kidney disease | <https://doi.org/10.1002/14651858.CD006763.pub2> | 2010 | Diabetes |
| Momordica charantia for type 2 diabetes mellitus | <https://doi.org/10.1002/14651858.CD007845.pub3> | 2012 | Diabetes |
| Cinnamon for diabetes mellitus | <https://doi.org/10.1002/14651858.CD007170.pub2> | 2012 | Diabetes |
| Enhanced glucose control for preventing and treating diabetic neuropathy | <https://doi.org/10.1002/14651858.CD007543.pub2> | 2012 | Diabetes |
| Computer-based diabetes self-management interventions for adults with type 2 diabetes mellitus | <https://doi.org/10.1002/14651858.CD008776.pub2> | 2013 | Diabetes |
| Sweet potato for type 2 diabetes mellitus | <https://doi.org/10.1002/14651858.CD009128.pub3> | 2013 | Diabetes |
| Vitamin B and its derivatives for diabetic kidney disease | <https://doi.org/10.1002/14651858.CD009403.pub2> | 2015 | Diabetes |
| Zinc supplementation for the prevention of type 2 diabetes mellitus in adults with insulin resistance | <https://doi.org/10.1002/14651858.CD005525.pub3> | 2015 | Diabetes |
| Intensive versus conventional glycaemic control for treating diabetic foot ulcers | <https://cochranelibrary.com/cdsr/doi/10.1002/14651858.CD010764.pub2/full> | 2016 | Diabetes |
| Self management interventions for type 2 diabetes in adult people with severe mental illness | <https://doi.org/10.1002/14651858.CD011361.pub2> | 2016 | Diabetes |
| Diet, physical activity or both for prevention or delay of type 2 diabetes mellitus and its associated complications in people at increased risk of developing type 2 diabetes mellitus | <https://doi.org/10.1002/14651858.CD003054.pub4> | 2017 | Diabetes |
| Effect of the treatment of Type 2 diabetes mellitus on the development of cognitive impairment and dementia | <https://doi.org/10.1002/14651858.CD003804.pub2> | 2017 | Diabetes |
| Acetyl-L-carnitine for the treatment of diabetic peripheral neuropathy | <https://cochranelibrary.com/cdsr/doi/10.1002/14651858.CD011265.pub2/full> | 2019 | Diabetes |
| Resveratrol for adults with type 2 diabetes mellitus | <https://doi.org/10.1002/14651858.CD011919.pub2> | 2020 | Diabetes |
| Nutritional interventions for treating foot ulcers in people with diabetes | <https://doi.org/10.1002/14651858.CD011378.pub2> | 2020 | Diabetes |
| Non-nutritive sweeteners for diabetes mellitus. | <https://doi.org/10.1002/14651858.CD012885.pub2> | 2020 | Diabetes |
| **Chronic respiratory disease** | | | |
| Dietary marine fatty acids (fish oil) for asthma in adults and children | <https://doi.org/10.1002/14651858.CD001283> | 2000 | Chronic respiratory disease |
| Tartrazine exclusion for allergic asthma | <https://doi.org/10.1002/14651858.CD000460> | 2001 | Chronic respiratory disease |
| Calorie controlled diet for chronic asthma | <https://doi.org/10.1002/14651858.CD004674.pub2> | 2003 | Chronic respiratory disease |
| Selenium supplementation for asthma | <https://doi.org/10.1002/14651858.CD003538.pub2> | 2004 | Chronic respiratory disease |
| Mono and multifaceted inhalant and/or food allergen reduction interventions for preventing asthma in children at high risk of developing asthma | <https://doi.org/10.1002/14651858.CD006480.pub2> | 2009 | Chronic respiratory disease |
| Caffeine for asthma | <https://doi.org/10.1002/14651858.CD001112.pub2> | 2010 | Chronic respiratory disease |
| Dietary sodium manipulation and asthma | <https://doi.org/10.1002/14651858.CD000436.pub3> | 2011 | Chronic respiratory disease |
| Nutritional supplementation for stable chronic obstructive pulmonary disease | <https://doi.org/10.1002/14651858.CD000998.pub3> | 2012 | Chronic respiratory disease |
| Weight loss interventions for chronic asthma | <https://doi.org/10.1002/14651858.CD009339.pub2> | 2012 | Chronic respiratory disease |
| Monosodium glutamate avoidance for chronic asthma in adults and children | <https://doi.org/10.1002/14651858.CD004357.pub4> | 2012 | Chronic respiratory disease |
| Vitamin C for asthma and exercise-induced bronchoconstriction | <https://doi.org/10.1002/14651858.CD010391.pub2> | 2013 | Chronic respiratory disease |
| Vitamins C and E for asthma and exercise-induced bronchoconstriction | <https://doi.org/10.1002/14651858.CD010749.pub2> | 2014 | Chronic respiratory disease |
| Vitamin D for the management of asthma | <https://doi.org/10.1002/14651858.CD011511.pub2> | 2016 | Chronic respiratory disease |
| Fluid restriction for treatment of preterm infants with chronic lung disease | <https://doi.org/10.1002/14651858.CD005389.pub2> | 2017 | Chronic respiratory disease |
| **Overweight & Obesity** | | | |
| Low glycaemic index or low glycaemic load diets for overweight and obesity | <https://doi.org/10.1002/14651858.CD005105.pub2> | 2007 | Overweight and obesity |
| Chitosan for overweight or obesity | <https://doi.org/10.1002/14651858.CD003892.pub3> | 2008 | Overweight and obesity |
| Green tea for weight loss and weight maintenance in overweight or obese adults | <https://doi.org/10.1002/14651858.CD008650.pub2> | 2012 | Overweight and obesity |
| Interactive computer-based interventions for weight loss or weight maintenance in overweight or obese people | <https://doi.org/10.1002/14651858.CD007675.pub2> | 2012 | Overweight and obesity |
| Chromium picolinate supplementation for overweight or obese adults | <https://doi.org/10.1002/14651858.CD010063.pub2> | 2013 | Overweight and obesity |
| Transtheoretical model stages of change for dietary and physical exercise modification in weight loss management for overweight and obese adults | <https://doi.org/10.1002/14651858.CD008066.pub3> | 2014 | Overweight and obesity |
| Parent-only interventions for childhood overweight or obesity in children aged 5 to 11 years | <https://cochranelibrary.com/cdsr/doi/10.1002/14651858.CD012008/full> | 2015 | Overweight and obesity |
| Effects of total fat intake on body weight | <https://doi.org/10.1002/14651858.CD011834> | 2015 | Overweight and obesity |
| Diet, physical activity, and behavioural interventions for the treatment of overweight or obesity in preschool children up to the age of 6 years | <https://doi.org/10.1002/14651858.CD012105> | 2016 | Overweight and obesity |
| Diet, physical activity and behavioural interventions for the treatment of overweight or obese adolescents aged 12 to 17 years | <https://doi.org/10.1002/14651858.CD012691> | 2017 | Overweight and obesity |
| Diet, physical activity and behavioural interventions for the treatment of overweight or obese children from the age of 6 to 11 years | <https://doi.org/10.1002/14651858.CD012651> | 2017 | Overweight and obesity |
| Interventions to change the behaviour of health professionals and the organisation of care to promote weight reduction in children and adults with overweight or obesity | <https://doi.org/10.1002/14651858.CD000984.pub3> | 2017 | Overweight and obesity |
| Effects of total fat intake on bodyweight in children | <https://doi.org/10.1002/14651858.CD012960.pub2> | 2018 | Overweight and obesity |
| Physical activity, diet and other behavioural interventions for improving cognition and school achievement in children and adolescents with obesity or overweight | <https://doi.org/10.1002/14651858.CD009728.pub4> | 2018 | Overweight and obesity |
| Interventions for preventing obesity in children | <https://doi.org/10.1002/14651858.CD001871.pub4> | 2019 | Overweight and obesity |
| Strategies to improve the implementation of healthy eating, physical activity and obesity prevention policies, practices or programmes within childcare services | <https://cochranelibrary.com/cdsr/doi/10.1002/14651858.CD011779.pub3/full> | 2020 | Overweight and obesity |
| Taxation of the fat content of foods for reducing their consumption and preventing obesity or other adverse health outcomes | <https://doi.org/10.1002/14651858.CD012415.pub2> | 2020 | Overweight and obesity |
| Taxation of unprocessed sugar or sugar-added foods for reducing their consumption and preventing obesity or other adverse health outcomes | <https://doi.org/10.1002/14651858.CD012333.pub2> | 2020 | Overweight and obesity |
| Effects of total fat intake on body fatness in adults | <https://doi.org/10.1002/14651858.CD013636> | 2020 | Overweight and obesity |
| Caregiver involvement in interventions for improving children's dietary intake and physical activity behaviors | <https://doi.org/10.1002/14651858.CD012547.pub2> | 2020 | Overweight and obesity |
| **Unhealthy Diets** | | | |
| Policy interventions implemented through sporting organisations for promoting healthy behaviour change | <https://doi.org/10.1002/14651858.CD004809.pub3> | 2008 | Unhealthy diets |
| Interventions to enhance adherence to dietary advice for preventing and managing chronic diseases in adults | <https://doi.org/10.1002/14651858.CD008722.pub2> | 2013 | Unhealthy diets |
| Portion, package or tableware size for changing selection and consumption of food, alcohol and tobacco | <https://doi.org/10.1002/14651858.CD011045.pub2> | 2015 | Unhealthy diets |
| Targeted mass media interventions promoting healthy behaviours to reduce risk of non-communicable diseases in adult, ethnic minorities | <https://doi.org/10.1002/14651858.CD011683.pub2> | 2017 | Unhealthy diets |
| Strategies for enhancing the implementation of school-based policies or practices targeting risk factors for chronic disease | <https://doi.org/10.1002/14651858.CD011677.pub2> | 2017 | Unhealthy diets |
| Environmental interventions to reduce the consumption of sugar-sweetened beverages and their effects on health | <https://www.cochranelibrary.com/cdsr/doi/10.1002/14651858.CD012292.pub2/full> | 2019 | Unhealthy diets |
| Altering the availability or proximity of food, alcohol and tobacco products to change their selection and consumption | <https://doi.org/10.1002/14651858.CD012573.pub3> | 2019 | Unhealthy diets |
| Interventions for increasing fruit and vegetable consumption in children aged five years and under | <https://doi.org/10.1002/14651858.CD008552.pub7> | 2020 | Unhealthy diets |

Table S2: Summary of research recommendations from Cochrane nutrition reviews addressing cardiovascular diseases (n=58)

| NCD Grouping |  | Domain (EPICOT+ item) |  | Research recommendation(s) (#) |  | Example |
| --- | --- | --- | --- | --- | --- | --- |
| Cardiovascular Diseases |  | Population |  | - Populations or individuals at risk of cardiovascular diseases (8,26,30,50,64) - Individuals with hypertension ( (38,39,62,119,124,128) - People with peripheral arterial disease (13) - Younger or older individuals with a previous cerebral haemorrhage or TIA (11,87) - People with post-stroke dysphagia (15) - People with FH and their care givers(86) - Individuals with Type 2 diabetes (7,26) - Populations or individuals at low risk of cardiovascular disease (8,64) - Normotensive individuals (3,119) - Healthy individuals (115,126) - Gender (both sexes) (29,39) - Women ((2,65,116,130) - Older people (41) - Younger people (29) - Ethnicity (e.g. African, Asian, South Asian, native Hawaiian/other Pacific Islander populations) (41,38,48,130) - Participants across a wider range of selenium status(116) - People with low and high baseline polyunsaturated fatty acid intakes (2) - People with low and high baseline omega-6 intake (65) - Populations with low calcium intake (29) - Poor individuals, socially excluded people (41) |  | “More placebo-controlled trials are warranted to clarify whether calcium supplementation can reduce blood pressure in people with elevated blood pressure.” (39) |
|  |  | Setting |  | - Primary care and the workplace compared with hospital settings (134) - Low- and middle-income and developing countries (2,41,65) - Higher income countries (1,65) - International setting (38) - Population and community level in the United Kingdom healthcare and other settings (115) - Population level (e.g. workplace, institutional, regulatory) (5) |  | “Research in the setting in which dietary advice is given would inform us about the effectiveness of advice in primary care and the workplace compared with hospital settings.” (134) |
|  |  | Intervention |  | Diets and dietary patterns   - Reduced saturated fat diet (replacement of saturated fats with unsaturated fats; combined with lipid-lowering medication) (63,64) - Reduced trans-fat intake (64) - Increased polyunsaturated fat intake (combined with low trans-fat intake) (2) - Low-fat diet (126) - Cholesterol-lowering diet (86) - Low sodium dietary advice (5,62) - Low GI diet (26) - Vegan diet (118) - Intermittent fasting (8) - Weight-reducing diet (124)   Food groups   - Dietary advice to increased fruit and vegetable consumption (50) - Black and green tea (49) - Whole grain cereals (e.g. Oats) (76)   Single foods   - Fermented milk (with higher content and /or better bioavailability of the active peptides) (136) - Cocoa (119) - Fish (1)   Macronutrient supplements   - Creatine analogues (66) - Alpha-linolenic acid (ALA) (1) - Omega-3 PUFA (formulation, dosage) (24)   Micronutrient supplements   - Vitamin E (dosage) (78) - Vitamin K (51) - Vitamin C (7) - Calcium (39); supplemental or dietary calcium (daily intake of at least 1gram/day) (29) - Magnesium (39) - Potassium (17,37) - Potassium and magnesium (17) - Selenium (116)   Complementary or alternative supplements   - Garlic (69); garlic (dosage) (128) - Isoﬂavones (38) - Coenzyme Q10 (46,56); Coenzyme Q10 (dosage) (85)   Nutrition education and counselling   - Elements and mode of administration of dietary advice (e.g. length and frequency of contact; individual or group, behavioural therapy or instructional techniques and interactive computer programmes, leaflets) (134) - Non-individualised modes of dietary health promotion (116) - mHealth interventions (10)   Policies, strategies and programmes   - Food legislation policies (to alter fat contents of foods, improved labelling, pricing initiatives and improved availability of healthier foods) (63,64) - Voluntary and regulatory salt reduction ( e.g. reduction of salt in processed foods) (5) - Alcohol intake reduction strategies (3) - Health protection for primary prevention (i.e. ﬁscal and legislative approaches) (41) - Weight loss and weight maintenance programs (30) - Self-management programmes (ideal frequency, mode and duration of sessions for stroke) (47) - Swallowing therapy (key components) (15) |  | “Long term research to help us understand what types of unsaturated fats are most useful in the diet when replacing saturated fats (monounsaturated fats, polyunsaturated fats and the relevant speciﬁc fatty acids) are urgently needed.” (10) |
|  |  | Comparison |  | - Placebo (24,37-39,78,85,113,128) - Usual/standard care or control (15,29,86,126) - Vitamin E versus placebo and another treatment (78) - Dietary intake of omega-3 PUFAs versus supplemental intake of omega 3 PUFAs (24) - Dietary intake of calcium versus supplemental intake of calcium (29) - Soluble versus insoluble fibre intake (52) - Dietary intake of fibre versus supplemental intake of fibre (52) - Homocysteine‐lowering interventions at high doses versus homocysteine‐lowering interventions at low doses (88) - Homocysteine‐lowering interventions and antihypertensive medication versus antihypertensive medication (88) |  | “A three-arm or factorial trial comparing vitamin E to another treatment as well as to placebo would be useful.” (78) |
|  |  | Outcome |  | - Mortality (1,5,8,24,48,49,62,76,78,85,123,124,128) - Morbidity (5,24,48,62,124,128) - Cardiovascular outcomes (1,8,26,39,64,116,118) - Cardiovascular events (8,24,41,46,49,50,51,52,63,65,76,85,113) - Cardiovascular disease risk factors (7,26,51,52,117,118) - Diabetes risk (65,115) - Hormonal and lipid outcomes (1,5,115) - Blood pressure (1,5,17,29,39,54) - Ankle brachial pressure index(ABI)(24) - Vasoconstriction (29) - Amputation rate (78) - Vascular surgery (78) - Time to healing (leg ulcers) (145) - Chest infection, pneumonia or signs of aspiration (15) - Heart failure hospitalisation (10) - Heart failure medication adherence (10) - Stroke (8), stroke recovery, recurrence (117) - Length of hospital stay (124) - Dietary sodium intake (94) - Patient satisfaction (134) - Quality of life or health related quality of life ,) (3,7,10,15,24,46,47,49,51,115,117) - Walking distance (24) - Adverse events (3,24,29,46,51,85,113,117,124,128) |  | “Trials utilising quality of life outcomes or cost-effectiveness evaluation are lacking.” (115) |
|  |  | Study Design |  | Design of primary studies   - Randomised Controlled trials (RCTs) (2,3,5,17,23,29,34,37,39,41,46, 48,49,51,56,62,63,69,85,86,88,115,116,119, 124,126,128,134,137) - Economic data /economic evaluation /cost effectiveness studies (7,34,41,46,47,49,51,115,116) - Random sequence generation and treatment allocation (7,15,37-39,63,65,78,145) - Blinding (7,15,37-39,63,65,145) - ITT analysis (39,63,78,145) - High quality randomised controlled trials (5,7,15,17,26,30,37,39,52,63,65,76,85,90,113,115,117,134,137,145) - A three-arm or factorial trial (78) - Larger trials or adequately powered (1,5,15,17,26,29,30,37-39,65,66,69,76,78,85,86,88,90,113,117,118,128,130,145) - Multicentre trials (15,30,86,113,145) - Qualitative studies(41) - Empirical studies (94) - Well-designed prospective population studies (48) - Attrition (15) - PROBE study (8) - Subgroup analysis (29)   Reporting by primary studies   - Adopting the CONSORT statement/guidelines (117,145) |  | “Further large and high-quality trials of ALA carried out in lower and higher income countries and that assess baseline ALA intake and use biomarkers to assess compliance would be helpful to clarify the cardiovascular effects of ALA. “(49) |
|  |  | Time frame |  | - Long-term trials (3,5,8,24,26,29,30,34,37-39,48-50,52,62-65,76,90,113,115,119,124,126) - Long-term effect of weight loss on stroke incidence (30) - Long enough time span to detect any long term effects on blood pressure (17) - Long-term effects of black and green tea interventions (49) - Sufﬁcient duration of length of follow-up (69,145) - Long-term follow-up data (124) |  | “They should enrol a large number of participants and have long enough follow-up to allow detection of any meaningful long-term effects of magnesium supplementation.” (39) |

Abbreviations: ALA, Alpha-linolenic acid; CONSORT, Consolidated Standards for Reporting of Trials; FH, familial hypercholesterolemia; omega-3 PUFAs, omega polyunsaturated fatty acids.

### ^#^citation number from supplemental reference list

Table S3: Summary of research recommendations from Cochrane nutrition reviews addressing cancer (n=26)

| NCD Grouping |  | Domain (EPICOT+ item) |  | Research recommendations (#) |  | Example |
| --- | --- | --- | --- | --- | --- | --- |
| Cancer |  | Population |  | - Healthy individuals (142) - Young people (19) - Men (19) - Individuals with low Vitamin D status (19) - Subgroups with baseline selenium exposure levels and genetic factors (138) - Children (19,27,108,140) - Non-Chinese population (73) - Individuals living with cancer (142,22) - Individuals with different types of cancer (35), other than breast cancer (22) - Individuals with head and neck cancer (104) - Young people with cancer (140) - Carers of people with cancer(140) - Individuals with advanced cancer and individuals with moderate to severe fatigue (16) - Homogeneous study populations (anticancer treatment received, disease stage specified, type of cancer) (31,73,108,127) - Malnourished populations (67), following surgery (21) - Different sub-groups of individuals living with endometrial cancer (e.g. stage of disease and timing of diagnosis) (77) - Individuals with mild to moderate diarrhoea (141) - Overweight & obese breast cancer survivors (125) |  | “Future trials should be RCTs performed in homogeneous study populations (e.g. with regards to anticancer treatment received).” (31) |
|  |  | Intervention |  | Diets and dietary patterns   - Dietary fibre (at least 30 to 40 mg per day) (149) - Weight-loss interventions (different dietary modification regimes) (77,125)   Single foods   - Green tea (low to moderate dosage) (44)   Macronutrient supplements or complete nutrition formulas   - EPA (more palatable formulations) (35) - Nutritional support (140) - Nutritional/dietary interventions (22,27), oral and enteral support pre- and post-operatively (21) - Different modalities of feeding (104) - Immunonutrition (67)   Micronutrient supplements   - Selenium (18); Sodium selenite (optimal dosage) (32) - Calcium (optimal dosage) (142) - Vitamin D (higher dosages) (19) - Iron (optimal dosage) (96) - Retinoic acid (108)   Complementary or alternative supplements   - Lycopene (68) - Flavonoid (standard dosage) (72) - Probiotics (141)   Nutritional education and counselling   - Educational interventions with different delivery options and their timing and format (16,21) |  | “Future trials should aim for the intervention group to have that level of intake (30 to 40 mg per day of fibre) and preferably higher.” (149) |
|  |  | Comparison |  | - Placebo (35, 72) |  | “There is need to conduct good quality large scale randomised controlled trials using EPA compared to placebo with different cancer types.” (35) |
|  |  | Outcomes |  | - Adverse effects and/or events (18,68,96,108); safety of intervention (140); risk-benefit balance (142) - Diagnosis of cancer (68,138) - Colorectal cancer (preferred endpoint) (149) - Mortality (68,96,22) - Survival (35,73,77,108,125,127) - Morbidity (22) - Cancer recurrence (22,125) - PSA levels (68) - Biomarker outcomes (125) - Quality of life (19,21,108,127) - Fatigue measures (16) - Work outcomes (16) - Dietary intake (27) - Body composition (21,22,27) - Cardiovascular & metabolic risk (27) - Patient reported outcomes (21) |  | “Future trials on the use of retinoic acid (either with or without other treatments like anti-GD2) after autologous stem cell transplant for children with high-risk neuroblastoma should be RCTs focusing on survival, (late) adverse effects, and quality of life.” (108) |
|  |  | Study Design |  | Design of primary studies   - Well-designed/High quality Randomised controlled trials (RCTs) (16,18,19,31,32,35,44,67,68,72,73,77,79,127,140,149) - Adequately powered and/or larger RCTs (16,31,35,44,67,77,79,108,140,141,142) - Multicentre trials (142,127) - Randomised Controlled Trials (RCTs) (18,19,96,27,108,77,21,125) (18,19,21,27,77,96,108) - Cost-effectiveness data / studies (16,19,68) - Adopting SPIRIT guidelines (19) - Explicitly explaining of trial methodology (enrolment, sequence generation, allocation concealment, randomisation, ITT analysis (73) and blinding (22,27,67,73) - Minimize biases (141 - Observational studies (138), non-experimental cohort studies (44), qualitative studies (127)   Reporting by primary studies   - Adopting/reporting according to the CONSORT statement and guidelines (16,18,19,35,79,141) - Standardised and consistent reporting of results (140), adequate reporting of completed, ongoing and future trials (32) |  | “There is need for a well-designed, high methodological quality, randomised controlled trial to investigate the effectiveness lycopene for the prevention of prostate cancer.” (68) |
|  |  | Timeframe |  | - Long period of follow-up / duration (19,22,44,77,79,96,108,149) |  | “RCTs should be performed in homogeneous study populations (like stage of disease) and have a long-term follow-up.” (108) |

### Abbreviations: CONSORT, Consolidated Standards for Reporting of Trials; EPA, Eicosapentaenoic acid; PSA, Prostate specific antigen.

### ^#^citation number from supplemental reference list

###

Table S4: Summary of research recommendations from Cochrane nutrition reviews addressing diabetes (n=24)

| NCD Grouping |  | Domain (EPICOT+ item) |  | Research recommendation (#) |  | Example |
| --- | --- | --- | --- | --- | --- | --- |
| Diabetes |  | Population |  | - Persons not currently overweight (103) - People with diabetes (70) - People with type 1 diabetes (23,120,121) - People with type 2 diabetes (23,102,105,107,121) - Children with diabetes (80,132) - Individuals with early development or different stages of diabetic kidney disease (114,129) - Genetic subgroups of the population at risk most susceptible to dietary intervention (111) - Participants with a higher mean baseline HbA1c greater than 7-8% (40,107) - People with impaired fasting glucose (55) |  | “There currently persists a research gap in the literature that investigates the effect of cinnamon in young children.” (80) |
|  |  | Intervention |  | Diets and dietary patterns   - Modest, sustained salt reduction (129) - Restricted protein diet (0.8 g /kg/day, with chicken and ﬁsh instead of red meat; or 1 g/kg/day, with vegetarian diet) (120) - Low-fat/high carbohydrate diet, modiﬁed-fat diet, restricted protein diet (102) - Low glycaemic index diets (132)   Food groups   - Whole grain foods (111)   Single foods   - Sweet potato (different varieties) (105) - Momordica charantia/ bitter gourd (use of different parts of the vegetable) (106)   Macronutrient supplements   - Omega-3 PUFAs (53)   Micronutrient supplements   - Vitamin B (optimal dosage) (114)   Complementary or alternative supplements   - Cinnamon (other species, different parameters of administration, extraction and preparation) (80) - Acetyl-L-carnitine (121) - Resveratrol (not combined with other plant polyphenols) (71)   Nutritional education and counselling   - Individual diabetes patient education(40) - Frequency and style of dietary advice (addition of behaviour modiﬁcation or not) (102) - Behaviour change techniques (107)   Policies, strategies and programmes   - Diet plus physical activity (55) - Exercise with reduced energy diet (102) - Sustained interventions for weight loss (103) |  | “Future research should explore other species of cinnamon and different parameters of administration, extraction and preparation.” (80) |
|  |  | Comparison |  | - Usual diet (unrestricted protein) or vegetarian diet with no restriction ( 120) - Group diabetes education(40) - Placebo (114) - Losartan (114) - Intensive vs conventional glycaemic control (42) - Placebo (121) |  | “There is a lack of evidence of short-term or long-term benefits of vitamin B compared to placebo or losartan on clinical and biochemical outcomes.” (114) |
|  |  | Outcome |  | - Mortality (36,93,102) - Incidence of type 2 diabetes mellitus (36) - Anti-diabetic medication (Change or delay in onset, compliance) (102) - Glycaemic control (132) - Complications of type 2 diabetes or diabetes (36,55,82,93,105) - Neuropathy and pain, impairment/disability, sensory function (23,121) - Amputation (42) - Wound healing (42,98) - Kidney function (GFR, urinary albumin) (120) - Insulin resistance (36) - Cardiovascular risk markers and risk reduction (53,107,120) - Weight loss (103,107) - Physical activity (107) - Adverse events (82,93,98,105,132) and safety (114) - Quality of life (36,82,93,98,102,103,120,121,122,132) - Depression (107) - Cognitive function (122) - Socio-economic status (36,93,71,82) - Product acceptability (98) |  | “Outcomes should include all of glomerular ﬁltration rate (GFR), quality of life, cost-effectiveness and cardiovascular risk factors such as lipids. “(120) |
|  |  | Study Design |  | Design of primary studies   - Economic evaluation or cost-effectiveness studies (80,98,103,105,107,120) - RCTs (36,40,42,53,55,70,80,82,93,105,111,114,120) - High quality studies (40,42,53,70,71,80,82,93,98,102,105,106,111,114) - with (allocation concealment, minimization of attrition, follow-up of dropouts, comparison of dropouts to completers at baseline, and ITT analysis (103). - Larger number or adequately powered studies (40,42,53,71,80,82,98,105,106,121) - Observational studies (105,106) - Evidenced and scientific based interventions following systematic and scientific design; explicitly 'prescribe' interventions for trials (107)   Reporting by primary studies   - Make use of one of the several reporting standards for clinical trials (80) - Clarity on reporting of adverse effects (105) - Future publications should ensure that the theoretical basis, active ingredients (behaviour change techniques) and doses of these ingredients (frequency of behaviour change techniques) are clearly described in published protocols and final reports. (93) |  | “In future, high quality RCTs are needed to establish efﬁcacy and safety proﬁle of vitamin B therapy especially treatment used in addition to the ﬁrst line treatment.” (114) |
|  |  | Time Frame |  | - Long-term studies (40,53,71,102,103,105,107,111,120,132)   Outcome measures that capture short- and long-term outcomes (80)   - Short-term or long-term benefits (114) |  | “Studies with longer follow-up are needed to determine the long-term impact on health outcomes of these interventions and look for evidence of harm.” (107) |

Abbreviations: GFR, Glomerular filtration rate; HbA1c, Haemoglobin A1c; omega-3 PUFAs, omega polyunsaturated fatty acids.

^#^citation number from supplemental reference list

### Table S5: Summary of research recommendations from Cochrane nutrition reviews addressing chronic respiratory diseases (n=14)

| NCD Grouping |  | Domain (EPICOT+ item) |  | Research recommendation (#) |  | Example |
| --- | --- | --- | --- | --- | --- | --- |
| Chronic respiratory diseases |  | Population |  | - People with chronic asthma (25) - Infants (from birth with at least six years of follow up) (84) - Patients with different levels of asthma severity (143) - Habitual consumers and non-consumers of caffeine (143) - Well controlled asthmatics on anti-inflammatory agents (143) - Adults and children with exercise induced bronchoconstriction/asthma (97,110,146) - People with chronic obstructive pulmonary disease (43) - Children (4,97,150) - Adolescents (4) - Vitamin D deficient children and adults who experience recurrent severe exacerbations (94) |  | “Further studies should examine whether weight reduction leads to asthma control in people with chronic asthma.” (25) |
|  |  | Setting |  | - Low-income countries (4) |  | “There is also a need for these well designed studies in children and adolescents, as well as in low-income countries such as Africa. “(4) |
|  |  | Intervention |  | Diets and dietary patterns   - Dietary reduction of calorie intake (25) - Dietary sodium manipulation (110) - Mono and multifaceted inhalant and/or food allergen reduction strategies (84) - Role of tartrazine and other food additives (12)   Single foods   - Fish (131)   Micronutrient supplements   - Vitamin C for exercise-induced asthma (97) - Vitamin C and E (dosage) (146) - Vitamin D (dosage) (43,94)   Policies, strategies and programmes   - Weight loss interventions (4) |  | “There is also a need for longer intervention as well as follow-up durations to evaluate the effect of sustained measures to achieve weight loss and to determine if these effects are still significantly present after a considerable period of time.” (4) |
|  |  | Comparison |  | - Mono versus multifaceted allergen reduction strategies (84) - Vitamin C (versus vitamins C and E) (146) - Vitamin E (versus vitamins C and E) (146) - Placebo (97,146,150) |  | “There is also a need for direct head-to-head comparative trials of mono and multifaceted allergen reduction strategies.” (84) |
|  |  | Outcome |  | - Survival (43) - Asthma exacerbation rates (40,43,94,97,131,146) - Asthma diagnosis, symptoms and control (4,12,84,131) - Use of rescue medication (4,97) - Bronchoconstriction, bronchodilation (143,146) - Hospital utilization or admission (4,43,131) - Patients’ perception (caffeine) (143) - Lung function (measurement and recording of symptoms) (12,25,4,97,146) - Anthropometric measures, muscle strength (43) - Adverse effects (4,97,146)   Quality of life (4,25,43,97,131,143,146)   - Exercise tolerance (146) - Impact on work and school (146) |  | “Future randomised placebo control trials should include the following outcomes robust symptom recording, HRQL , exacerbation rates and adverse effects.” (146) |
|  |  | Study design |  | Design of primary studies   - Larger studies or adequately powered studies (9,12,14,25,97,110,131,146,150) - RCTs (4,9,12,14,84,94,97,146,150) - High Quality Trials (4,97,146) - Direct head-to-head comparative trials of mono and multifaceted allergen reduction strategies (84) - Economic evaluation/cost effectiveness studies (84) - Physiological studies (14) - Masking (14), Blinding and allocation concealment (4,12,84,97) |  | “Larger randomised trials are required in further research for selenium treatment for asthma.” (9) |
|  |  | Timeframe |  | - Long term follow up (4,25,84) |  | “There is also a need for longer intervention as well as follow-up durations to evaluate the effect of sustained measures to achieve weight loss and to determine if these effects are still significantly present after a considerable period of time.” (4) |

^#^citation number from supplemental reference list

Table S6: Summary of research recommendations from Cochrane nutrition reviews addressing obesity and overweight (n=20)

| NCD Grouping |  | Domain (EPICOT+ item) |  | Research recommendation (#) |  | Example |
| --- | --- | --- | --- | --- | --- | --- |
| Overweight and Obesity |  | Population |  | - Children and adolescents with obesity or overweight (89) - Overweight and obese adults (45,92) - Parents in pediatric obesity intervention studies (95) - Parents who are obese and their children (6) - Ethnicity, e.g. children from minority ethnic groups (95) - Patients and healthcare professionals (45) - Children and adolescents (20,60,89,101) |  | “More evidence is needed to determine effective interventions in young children, particularly those aged 0-3 years, and adolescents.” (20) |
|  |  | Setting |  | - Low- and middle-income countries (20,45,89,95,99,101) - Transitional or developing countries where fat intakes of between 25-30% or greater than 30% of energy (60,61) - High income countries (99) - Countries that tax unprocessed sugar or sugar-added foods (109) - Healthcare or clinical setting (45, 89) - Community based, socially deprived environments (89) - Faith-based settings (20) |  | “We do recommend that further research in the early years and adolescence is conducted, and that research should include a wider range of community settings (including faith-based settings).” (20) |
|  |  | Intervention |  | Diets and dietary patterns   - Different diet-only interventions (28) - Low fat intakes (long term safety) (101)   Single foods   - Green tea (75)   Complementary or alternative supplements   - Chitosan (dosage, composition) (74) - Chromium picolinate (135)   Nutrition education and counselling   - Transtheoretical model stages of change (TTM SOC) (92) - Educational interventions for healthcare professionals (e.g. interactive online courses and learning materials) (45)   Policies, strategies and programmes   - Interactive computer-based interventions (144) - E-health systems (using distal measuring devices for weight management) (45) - Use of smartphone functions (weight management) (45) - Health-promoting initiatives targeting excessive weight gain (148) - Multicomponent obesity prevention and treatment programmes (including physical activity intervention components) (89) - Care-giver involved interventions (to improve children’s dietary intake and physical activity) (99) - Taxation of foods containing fat/saturated fat (81) - Taxation of sugar-added foods or unprocessed sugar (109) |  | “There is a need for new longer‐term studies that are designed specifically to answer the question of whether lower fat compared to higher fat diets are safe and effective for preventing abnormal weight gain, overweight or obesity in the long term in generally healthy children with healthy bodyweights.” (101) |
|  |  | Comparison |  | - Higher fat diets (101) - Standard care or practice (45,89) |  | “Future studies should ensure that innovative interventions are always compared to ’standard care’.(45) |
|  |  | Outcome |  | - Prevalence of overweight (20) - BMI (or zBMI) (20) - Mortality (74) - Morbidity (74,135,95,45) - Dietary outcomes (6) - Physical activity (6) and behaviour change (95) - Cognition and school achievement (89) - Self-esteem (6) - Quality of life (6,74,75,83,92,95,133,135) - Adherence (144) - Adverse effects (6,28,75,133) |  | “Future studies should include outcomes such as health-related quality of life and adverse effects.” (75) |
|  |  | Study Design |  | Design of primary studies   - Randomised Controlled trials (6,45,60,61,74,89,92,95,99,101,135) - Economic Evaluation/cost effective studies (6,20,45,74,83,99) - ITT and per protocol (PP) analysis (75) - Large/adequately powered studies (89,92,135) - Use a protocol when conducting and reporting research (92) - Study designs other than randomised controlled trials (95) - Qualitative research (95) - Prospective cohort studies (81,101) - Methods to impute missing outcome data (89) - Natural, experimental studies (81) - High Quality studies (81,61) (randomisation, allocation concealment, attrition, blinding and intention to treat analysis and power calculations) (6,45,60,74,92,99,101)   Reporting by primary studies   - Adopting CONSORT guidelines (45,75,99) - Using recommended reporting guidelines (101) - Collect information at baseline on gender and other PROGRESS factors, but also to analyse the effect of the intervention by these factors (20,99) - Use of comprehensive theoretical frameworks could assist in considering a broad range of implementation barriers and designing appropriate support strategies (148) - Need to specify intervention content using established frameworks, relying on internationally recognized terms and definitions (99) |  | “Future studies should follow a standard reporting format, such as CONSORT, to ensure that all details of the study are available for assessment and perform an ITT and per protocol (PP) analysis to account for drop-outs after randomisation.” (75) |
|  |  | Time frame |  | - Longer-term follow-up studies (20,74,75,89,92,95,101,133,144) |  | “We suggest that interventions and strategies to prevent obesity in children should include follow-up over several years.” (20) |

Abbreviations: BMI, Body mass index; CONSORT, Consolidated Standards for Reporting of Trials; PROGRESS, Place, Race, Occupation, Gender, Religion, Education, Socio‐economic status; factors; zBM, Body mass index z-score

#citation number from supplemental reference list

Table S7: Summary of research recommendations from Cochrane nutrition reviews addressing unhealthy diets (n=8)

| NCD Grouping |  | Domain (EPICOT+ item) |  | Research recommendation (#) |  | Example |
| --- | --- | --- | --- | --- | --- | --- |
| Unhealthy Diets |  | Population |  | - Children from low-income, minority or indigenous communities (57) - Culturally diverse backgrounds (e.g. Indigenous people) (112) - Ethnic minority populations (with different first languages to the general population) (100) |  | “The investigation of the impact of interventions for children from low-income, minority or indigenous communities…” (57) |
|  |  | Setting |  | - Sports settings (e.g. sports clubs) (57,112) - Field setting (58) - LMICs (58) - Different media structures, economic environments (100) - Schools (147) - Preschools, play-groups (57,147) - Co-operatives (57) |  | “The key research implication of this review is that more high‐quality studies of both availability and proximity interventions in field settings are needed.” (58) |
|  |  | Intervention |  | Policies, strategies and programmes   - Targeted mass media interventions (diet, physical activity, alcohol and tobacco use) (100) - Smaller‐sized portions, packages, individual units and tableware for consumption of food, alcohol (59) - Strategies for implementation of health promotion programs in schools (147) - Availability and proximity interventions (alcohol and tobacco products) (58) - Behavioural interventions delivered by health professionals, telephone- or computer-based programmes (57) |  | “Further new primary studies of the effects of exposure to larger versus smaller‐sized portions, packages, individual units and tableware on selection and consumption of food are needed.” (59) |
|  |  | Comparison |  | - General mass media interventions (diet, physical activity, alcohol and tobacco use) (100) - Larger‐sized portions, packages, individual units and tableware for consumption of food, alcohol (59) - Sporting organisation (matched by size, geographical area, demographics, etc) (112) - Control/usual care group (162) |  | “New studies should be designed to directly compare the effects of targeted versus general mass media interventions with equal intensities and broader frames. “(100) |
|  |  | Outcome |  | - Development of policies, implementation of policies and changes in individual behaviour relating to the particular policy (112) - Sun protection habits, alcohol use, smoking status, frequency of healthy eating (112) - Adherence (dietary advice) (162) - Perspectives from health professionals and clients about the interventions enhancing adherence (162) - Adverse effects (e.g. increased family grocery costs, parent self-esteem or sense of competence) (57,147) - Dietary intake (self-reported) (139) - Electronic sales data (139) |  | “…the investigation of potential adverse effects of interventions (e.g. increased family grocery costs, or adverse effects on parental self‐esteem or sense of competence) as a routine part of intervention trials.” (109) |
|  |  | Study Design |  | Design of primary studies   - Factorial design studies (for policy interventions) (112) - Repeated measurements before and after design (112) - Cluster design studies with adequate sample size per cluster (112) - Rigorous, High quality studies (33,58,59,100,109,139,) - Randomised controlled trials (33,100,109,147), cluster-RCTs (100) - Further studies designed to minimise bias (33,139) - Economic evaluation/cost effective studies (109,147) - Incorporation of logic models (147) - Larger/adequate sample sizes (139)   Reporting by primary studies   - Reporting should comply with CONSORT-SPI (59), including descriptions of interventions that allow for their replication. - Improved rigour and reporting (58,147) - Studies to assess and report the impact of interventions; Using TIPPME typology (58) |  | “Future trials should include examination of the cost-effectiveness of interventions found to be effective. “ (57) |
|  |  | Time frame |  | - Interventions with extended periods of follow-up (33,100,109,139) |  | Further studies with a long-term duration of more than 12 months, and a follow-up evaluation are needed(33). |

Abbreviations: CONSORT, Consolidated Standards for Reporting of Trials; LMICs, Low- and middle-income countries

^#^citation number from supplemental reference list

**Supplementary reference list**

1. Abdelhamid AS, Brown TJ, Brainard JS et al. (2020) Omega‐3 fatty acids for the primary and secondary prevention of cardiovascular disease. *Cochrane Database Syst Rev* **3:**CD003177.
2. Abdelhamid, A. S., Martin, N., Bridges, C. et al. (2018) Polyunsaturated fatty acids for the primary and secondary prevention of cardiovascular disease. *Cochrane Database Syst Rev* **7:** CD012345.
3. Acin, M. T., Rueda, J. R., Saiz, L. C. et al. (2020) Alcohol intake reduction for controlling hypertension. *Cochrane Database Syst Rev* **9:** CD010022*.*
4. Adeniyi FB, Young T (2012). Weight loss interventions for chronic asthma. *Cochrane Database Syst Rev* **7:** CD009339*.*
5. Adler AJ, Taylor F, Martin N et al. (2020) Reduced dietary salt for the prevention of cardiovascular disease. *Cochrane Database Syst Rev* **12:** CD009217.
6. Al‐Khudairy L, Loveman E, Colquitt JL et al. (2017) Diet, physical activity and behavioural interventions for the treatment of overweight or obese adolescents aged 12 to 17 years. *Cochrane Database Syst Rev* **6:** CD012691.
7. Al-Khudairy, L., Flowers, N., Wheelhouse, R. et al. (2017). Vitamin C supplementation for the primary prevention of cardiovascular disease. *Cochrane Database Syst Rev* **3:** CD011114.
8. Allaf, M., Elghazaly, H., Mohamed, O. G. et al. (2021) Intermittent fasting for the prevention of cardiovascular disease. *Cochrane Database Syst Rev* **1:** CD013496.
9. Allam MF, Lucena RA (2004). Selenium supplementation for asthma. *Cochrane Database Syst Rev* **2:** CD003538.
10. Allida, S., Du, H., Xu, X. et al. (2020) mHealth education interventions in heart failure. *Cochrane Database Syst Rev* **7:** CD011845.
11. Alvarez Campano, C. G., Macleod, M. J., Aucott, L. et al. (2019). Marine-derived n-3 fatty acids therapy for stroke. *Cochrane Database Syst Rev* **6:** CD012815.
12. Ardern K (2001). Tartrazine exclusion for allergic asthma. *Cochrane Database Syst Rev* ***4:*** CD000460.
13. Aung PP, Maxwell H, Jepson RG et al. (2007) Lipid‐lowering for peripheral arterial disease of the lower limb. *Cochrane Database Syst Rev* **4:** CD000123.
14. Barrington KJ, Fortin-Pellerin E, Pennaforte T (2017) Fluid restriction for treatment of preterm infants with chronic lung disease. *Cochrane Database Syst Rev* **2:** CD005389.
15. Bath PM, Lee HS, Everton LF (2018). Swallowing therapy for dysphagia in acute and subacute stroke. *Cochrane Database Syst Rev* **10:** CD000323.
16. Bennett S, Pigott A, Beller EM et al. (2016) Educational interventions for the management of cancer-related fatigue in adults. *Cochrane Database Syst Rev* **10:** CD008144.
17. Beyer FR, Dickinson HO, Nicolson D et al. (2006). Combined calcium, magnesium and potassium supplementation for the management of primary hypertension in adults. *Cochrane Database Syst Rev* **3**: CD004805.
18. Bjelakovic G, Nikolova D, Simonetti RG et al. (2008) Antioxidant supplements for preventing gastrointestinal cancers. *Cochrane Database Syst Rev* **3:** CD004183.
19. Bjelakovic G, Gluud LL, Nikolova D et al. (2014) Vitamin D supplementation for prevention of cancer in adults. *Cochrane Database Syst Rev* **6:** CD007469.
20. Brown T, Moore TH, Hooper L et al. (2019) Interventions for preventing obesity in children. *Cochrane Database Syst Rev* **7**: CD001871.
21. Burden S, Billson HA, Lal S et al. (2019) Perioperative nutrition for the treatment of bladder cancer by radical cystectomy. *Cochrane Database Syst Rev* **5:** CD010127**.**
22. Burden S, Jones DJ, Sremanakova J et al. (2019) Dietary interventions for adult cancer survivors. *Cochrane Database Syst Rev* 11. CD011287.
23. Callaghan BC, Little AA, Feldman EL et al. (2012) Enhanced glucose control for preventing and treating diabetic neuropathy. *Cochrane Database Syst Rev* **6**: CD007543.
24. Campbell A, Price J, Hiatt WR (2013). Omega‐3 fatty acids for intermittent claudication. *Cochrane Database Syst Rev* **7**: CD003833.
25. Cheng J, Pan T (2003) Calorie controlled diet for chronic asthma. *Cochrane Database Syst Rev* **2**: CD004674.
26. Clar C, Al‐Khudairy L, Loveman E et al. (2017) Low glycaemic index diets for the prevention of cardiovascular disease. *Cochrane Database Syst Rev* **7**: CD004467.
27. Cohen JE, Wakefield CE, Cohn RJ (2016). Nutritional interventions for survivors of childhood cancer. *Cochrane Database Syst Rev* **8**: CD009678.
28. Colquitt JL, Loveman E, O’Malley C et al. (2016) Diet, physical activity, and behavioural interventions for the treatment of overweight or obesity in preschool children up to the age of 6 years. *Cochrane Database Syst Rev* **3:** CD012105.
29. Cormick G, Ciapponi A, Cafferata ML, Belizán JM (2015) Calcium supplementation for prevention of primary hypertension. *Cochrane Database Syst Rev* **6**: CD010037.
30. Curioni C, André C, Veras R (2006) Weight reduction for primary prevention of stroke in adults with overweight or obesity. *Cochrane Database Syst Rev* **4**: CD006062.
31. Dalen EC van, Mank A, Leclercq E et al. (2016) Low bacterial diet versus control diet to prevent infection in cancer patients treated with chemotherapy causing episodes of neutropenia. *Cochrane Database Syst Rev* **4**: CD006247.
32. Dennert G, Horneber M. Selenium for alleviating the side effects of chemotherapy, radiotherapy and surgery in cancer patients. *Cochrane Database Syst Rev* **3**: CD005037.
33. Desroches S, Lapointe A, Ratté S et al. (2013) Interventions to enhance adherence to dietary advice for preventing and managing chronic diseases in adults. *Cochrane Database Syst Rev* **2**: CD008722.
34. Devi R, Singh SJ, Powell J (2015). Internet-based interventions for the secondary prevention of coronary heart disease. *Cochrane Database Syst Rev* **12**: CD009386.
35. Dewey A, Baughan C, Dean TP et al. (2007) Eicosapentaenoic acid (EPA, an omega‐3 fatty acid from fish oils) for the treatment of cancer cachexia. *Cochrane Database Syst Rev* **1**: CD004597.
36. Dib RE, Gameiro OL, Ogata MS et al. (2015) Zinc supplementation for the prevention of type 2 diabetes mellitus in adults with insulin resistance. *Cochrane Database Syst Rev* **5**: CD005525.
37. Dickinson HO, Nicolson D, Campbell F et al. (2006) Potassium supplementation for the management of primary hypertension in adults. *Cochrane Database Syst Rev* **3**: CD004641.
38. Dickinson HO, Nicolson D, Campbell F et al. (2006) Magnesium supplementation for the management of primary hypertension in adults. *Cochrane Database Syst Rev* **3**: CD004640.
39. Dickinson HO, Nicolson D, Cook JV et al. (2006) Calcium supplementation for the management of primary hypertension in adults. *Cochrane Database Syst Rev* **2**: CD 004639.
40. Duke S-AS, Colagiuri S, Colagiuri R (2009). Individual patient education for people with type 2 diabetes mellitus. *Cochrane Database Syst Rev* **1:** CD005268*.*
41. Ebrahim S, Taylor F, Ward K et al. (2011) Multiple risk factor interventions for primary prevention of coronary heart disease. *Cochrane Database Syst Rev* **1**: CD001561.
42. Fernando ME, Seneviratne RM, Tan YM et al. (2016) Intensive versus conventional glycaemic control for treating diabetic foot ulcers. *Cochrane Database Syst Rev* **1**: CD010764.
43. Ferreira IM, Brooks D, White J et al. (2012) Nutritional supplementation for stable chronic obstructive pulmonary disease. *Cochrane Database Syst Rev* **12:** CD000998.
44. Filippini T, Malavolti M, Borrelli F et al. (2020) Green tea (Camellia sinensis) for the prevention of cancer. *Cochrane Database Syst Rev*: **3:** CD005004.
45. Flodgren G, Gonçalves‐Bradley DC et al. (2017) Interventions to change the behaviour of health professionals and the organisation of care to promote weight reduction in children and adults with overweight or obesity. *Cochrane Database Syst Rev* **11**: CD010405.
46. Flowers N, Hartley L, Todkill D, et al. (2014) Co‐enzyme Q10 supplementation for the primary prevention of cardiovascular disease. *Cochrane Database Syst Rev* **12**:CD010405.
47. Fryer CE, Luker JA, Mcdonnell MN et al. (2016). Self management programmes for quality of life in people with stroke. *Cochrane Database of Systematic Reviews* **8**: CD010442.
48. Graudal NA, Hubeck‐Graudal T, Jurgens G (2017). Effects of low sodium diet versus high sodium diet on blood pressure, renin, aldosterone, catecholamines, cholesterol, and triglyceride. *Cochrane Database Syst Rev* **4**: CD004022.
49. Hartley L, Flowers N, Holmes J, et al. (2013) Green and black tea for the primary prevention of cardiovascular disease. *Cochrane Database Syst Rev* **6**: CD009934.
50. Hartley L, Igbinedion E, Holmes J et al. (2013) Increased consumption of fruit and vegetables for the primary prevention of cardiovascular diseases. *Cochrane Database Syst Rev* **6**: CD009874.
51. Hartley L, Clar C, Ghannam O et al. (2015) Vitamin K for the primary prevention of cardiovascular disease. *Cochrane Database Syst Rev* **9**:CD011148.
52. Hartley L, May MD, Loveman E et al. (2016). Dietary fibre for the primary prevention of cardiovascular disease. *Cochrane Database Syst Rev* **2**: CD011472.
53. Hartweg J, Perera R, Montori VM et al. (2008) Omega‐3 polyunsaturated fatty acids (PUFA) for type 2 diabetes mellitus. *Cochrane Database Syst Rev* **1**: CD003205.
54. He FJ, Li J, MacGregor GA. Effect of longer‐term modest salt reduction on blood pressure. *Cochrane Database Syst Rev* **4**: CD004937.
55. Hemmingsen B, Gimenez‐Perez G, Mauricio D et al. (2017) Diet, physical activity or both for prevention or delay of type 2 diabetes mellitus and its associated complications in people at increased risk of developing type 2 diabetes mellitus. *Cochrane Database Syst Rev* **12**: CD003054.
56. Ho, M. J., Li, E. C., Wright, J. M. (2016). Blood pressure lowering efficacy of coenzyme Q10 for primary hypertension. *Cochrane Database Syst Rev*: **3:** CD007435.
57. Hodder RK, O’Brien KM, Tzelepis F et al. (2020) Interventions for increasing fruit and vegetable consumption in children aged five years and under. *Cochrane Database Syst Rev* **5**: CD008552.
58. Hollands GJ, Carter P, Anwer S et al. (2019) Altering the availability or proximity of food, alcohol, and tobacco products to change their selection and consumption. *Cochrane Database Syst Rev* **8**: CD012573.
59. Hollands GJ, Shemilt I, Marteau TM et al. (2015) Portion, package or tableware size for changing selection and consumption of food, alcohol and tobacco. *Cochrane Database Syst Rev* **9**: CD011045.
60. Hooper L, Abdelhamid A, Bunn D et al. (2015) Effects of total fat intake on body weight. *Cochrane Database Syst Rev* **8**: CD011834.
61. Hooper L, Abdelhamid AS, Jimoh OF et al. (2020) Effects of total fat intake on body fatness in adults. *Cochrane Database Syst Rev* **6**: CD013636.
62. Hooper L, Bartlett C, Smith GD et al. (2004) Advice to reduce dietary salt for prevention of cardiovascular disease*. Cochrane Database Syst Rev* **1**: CD003656.
63. Hooper L, Martin N, Abdelhamid A et al. (2015) Reduction in saturated fat intake for cardiovascular disease. *Cochrane Database Syst Rev* **9**: CD011737.
64. Hooper L, Summerbell CD, Thompson R et al. (2012) Reduced or modified dietary fat for preventing cardiovascular disease. *Cochrane Database Syst Rev* **5**: CD002137.
65. Hooper L, Al-Khudairy L, Abdelhamid AS et al. (2018) Omega-6 fats for the primary and secondary prevention of cardiovascular disease. *Cochrane Database Syst Rev* **11**: CD011094.
66. Horjus DL, Oudman I, Montfrans GA van et al. (2011) Creatine and creatine analogues in hypertension and cardiovascular disease. *Cochrane Database Syst Rev* **11**: CD005184.
67. Howes N, Atkinson C, Thomas S et al. (2018), Lewis SJ. Immunonutrition for patients undergoing surgery for head and neck cancer. *Cochrane Database Syst Rev* **8**: CD010954*.*
68. Ilic D, Forbes KM, Hassed C. (2011) Lycopene for the prevention of prostate cancer. *Cochrane Database Syst Rev* **11**: CD008007.
69. Jepson RG, Kleijnen J, Leng GC. (2013) Garlic for peripheral arterial occlusive disease. *Cochrane Database Syst Rev* **4**: CD000095.
70. Jesus CCL de, Atallah ÁN, Valente O et al. (2008) Vitamin C and superoxide dismutase (SOD) for diabetic retinopathy. *Cochrane Database Syst Rev* **1**: CD006695.
71. Jeyaraman MM, Al-Yousif NSH, Singh Mann A et al. (2020) Resveratrol for adults with type 2 diabetes mellitus. *Cochrane Database Syst Rev* **1***:* CD011919*.*
72. Jin H, Leng Q, Li C. (2012) Dietary flavonoid for preventing colorectal neoplasms. *Cochrane Database Syst Rev* **8**: CD009350.
73. Jin X, Beguerie JR, Sze DM, Chan GC. *Ganoderma lucidum* (Reishi mushroom) for cancer treatment. *Cochrane Database Syst Rev* **4**: CD007731.
74. Jull AB, Mhurchu CN, Bennett DA et al. (2008) Chitosan for overweight or obesity. *Cochrane Database Syst Rev* **3:** CD003892.
75. Jurgens TM, Whelan AM, Killian L et al. (2012) Green tea for weight loss and weight maintenance in overweight or obese adults. *Cochrane Database Syst Rev* **12:** CD008650*.*
76. Kelly SA, Hartley L, Loveman E et al. (2017) Whole grain cereals for the primary or secondary prevention of cardiovascular disease. *Cochrane Database Syst Rev* **8:** CD005051.
77. Kitson S, Ryan N, Mackintosh ML et al. (2018) Interventions for weight reduction in obesity to improve survival in women with endometrial cancer. *Cochrane Database Syst Rev* **2***:* CD012513*.*
78. Kleijnen J, Mackerras D (1998). Vitamin E for intermittent claudication. *Cochrane Database Syst Rev* **1 :** CD000987.
79. Lawrie TA, Green JT, Beresford M et al. (2018) Interventions to reduce acute and late adverse gastrointestinal effects of pelvic radiotherapy for primary pelvic cancers. *Cochrane Database Syst Rev* **1**: CD012529*.*
80. Leach MJ, Kumar S. (2012) Cinnamon for diabetes mellitus. *Cochrane Database Syst Rev* **9**: CD007170.
81. Lhachimi SK, Pega F, Heise TL et al. (2020) Taxation of the fat content of foods for reducing their consumption and preventing obesity or other adverse health outcomes. *Cochrane Database Syst Rev* **9:** CD012415.
82. Lohner S, Kuellenberg de Gaudry D et al. (2020) Non-nutritive sweeteners for diabetes mellitus. *Cochrane Database Syst Rev* **5***:* CD012885*.*
83. Loveman E, Al-Khudairy L, Johnson RE et al. (2015) Parent-only interventions for childhood overweight or obesity in children aged 5 to 11 years. *Cochrane Database Syst Rev* **12:** CD012008.
84. Maas T, Kaper J, Sheikh A et al. (2009) Mono and multifaceted inhalant and/or food allergen reduction interventions for preventing asthma in children at high risk of developing asthma. *Cochrane Database Syst Rev* **3**: CD006480.
85. Madmani ME, Solaiman AY, Agha KT et al. (2014) Coenzyme Q10 for heart failure. *Cochrane Database Syst Rev* **6**: CD008684.
86. Malhotra A, Shafiq N, Arora A et al. (2014) Dietary interventions (plant sterols, stanols, omega‐3 fatty acids, soy protein and dietary fibers) for familial hypercholesterolaemia. *Cochrane Database Syst Rev* **6**: CD001918.
87. Manktelow BN, Potter JF (2009). Interventions in the management of serum lipids for preventing stroke recurrence. *Cochrane Database Syst Rev* **3**: CD002091.
88. Martí-Carvajal AJ, Solà I, Lathyris D et al. (2017). Homocysteine-lowering interventions for preventing cardiovascular events. *Cochrane Database Syst Rev* **8**: CD006612.
89. Martin A, Booth JN, Laird Y et al. (2018) Physical activity, diet and other behavioural interventions for improving cognition and school achievement in children and adolescents with obesity or overweight. *Cochrane Database Syst Rev* **3**: CD009728.
90. Martin N, Germanò R, Hartley L et al. (2015). Nut consumption for the primary prevention of cardiovascular disease. *Cochrane Database Syst Rev* **9**: CD011583.
91. Martineau AR, Cates CJ, Urashima M et al. (2016) Vitamin D for the management of asthma. *Cochrane Database Syst Rev* **9**: CD011511.
92. Mastellos N, Gunn LH, Felix LM et al. (2014) Transtheoretical model stages of change for dietary and physical exercise modification in weight loss management for overweight and obese adults. *Cochrane Database Syst Rev* **7**: CD008066.
93. Mcbain H, Mulligan K, Haddad M et al. (2016) Self management interventions for type 2 diabetes in adult people with severe mental illness. *Cochrane Database Syst Rev* **4**:CD011361*.*
94. Mclaren L, Sumar N, Barberio AM et al. (2016) Population-level interventions in government jurisdictions for dietary sodium reduction. *Cochrane Database Syst Rev* **9**: CD010166.
95. Mead E, Brown T, Rees K et al. (2017) Diet, physical activity and behavioural interventions for the treatment of overweight or obese children from the age of 6 to 11 years. *Cochrane Database Syst Rev* **6**: CD012651.
96. Mhaskar R, Wao H, Miladinovic B et al. (2016) The role of iron in the management of chemotherapy-induced anemia in cancer patients receiving erythropoiesis-stimulating agents. *Cochrane Database Syst Rev* **2**: CD009624.
97. Milan SJ, Hart A, Wilkinson M (2013) Vitamin C for asthma and exercise‐induced bronchoconstriction. *Cochrane Database Syst Rev* **10:** CD010391.
98. Moore ZEH, Corcoran MA, Patton D. (2020) Nutritional interventions for treating foot ulcers in people with diabetes. *Cochrane Database Syst Rev* 7: CD011378.
99. Morgan EH, Schoonees A, Sriram U et al. (2020) Caregiver involvement in interventions for improving children’s dietary intake and physical activity behaviors. *Cochrane Database Syst Rev* **1**: CD012547.
100. Mosdøl A, Lidal IB, Straumann GH et al. (2017) Targeted mass media interventions promoting healthy behaviours to reduce risk of non-communicable diseases in adult, ethnic minorities. *Cochrane Database Syst Rev* **2**: CD011683.
101. Naude CE, Visser ME, Nguyen KA et al. (2018) Effects of total fat intake on bodyweight in children. . *Cochrane Database Syst Rev* **7**: CD012960.
102. Nield L, Moore H, Hooper L et al. (2007) Dietary advice for treatment of type 2 diabetes mellitus in adults. *Cochrane Database Syst Rev* **3**: CD004097.
103. Norris SL, Zhang X, Avenell A et al. (2005) Long‐term non‐pharmacological weight loss interventions for adults with type 2 diabetes mellitus. *Cochrane Database Syst Rev* **2**: CD004095.
104. Nugent B, Lewis S, O’Sullivan JM (2013) Enteral feeding methods for nutritional management in patients with head and neck cancers being treated with radiotherapy and/or chemotherapy. *Cochrane Database Syst Rev* **1:** CD007904**.**
105. Ooi CP, Loke SC (2013) Sweet potato for type 2 diabetes mellitus. *Cochrane Database Syst Rev* **9:** CD009128*.*
106. Ooi CP, Yassin Z, Hamid TA (2012). Momordica charantia for type 2 diabetes mellitus. *Cochrane Database Syst Rev* **8**:CD007845*.*
107. Pal K, Eastwood SV, Michie S et al. (2013) Computer‐based diabetes self‐management interventions for adults with type 2 diabetes mellitus. *Cochrane Database Syst Rev* **3**: CD008776.
108. Peinemann F, Dalen EC van, Enk H et al. (2017) Retinoic acid postconsolidation therapy for high‐risk neuroblastoma patients treated with autologous haematopoietic stem cell transplantation. *Cochrane Database Syst Rev* **8**: CD010685.
109. Pfinder M, Heise TL, Hilton Boon M et al. (2020) Taxation of unprocessed sugar or sugar-added foods for reducing their consumption and preventing obesity or other adverse health outcomes. *Cochrane Database Syst Rev* ***8:*** CD012333.
110. Pogson Z, McKeever T. (2011) Dietary sodium manipulation and asthma. *Cochrane Database Syst Rev* 3: CD000436.
111. Priebe M, Binsbergen J van, Vos R de et al. (2008) Whole grain foods for the prevention of type 2 diabetes mellitus. *Cochrane Database Syst Rev* **1**: CD006061.
112. Priest N, Armstrong R, Doyle J et al. (2008) Policy interventions implemented through sporting organisations for promoting healthy behaviour change. *Cochrane Database Syst Rev* **3**: CD004809.
113. Qin Y, Niu K, Zeng Y et al. (2013) Isoflavones for hypercholesterolaemia in adults. *Cochrane Database Syst Rev* **6**: CD009518.
114. Raval AD, Thakker D, Rangoonwala AN et al. (2015) Vitamin B and its derivatives for diabetic kidney disease. *Cochrane Database Syst Rev* **1**: CD009403.pub2/full
115. Rees K, Dyakova M, Wilson N et al. (2013) Dietary advice for reducing cardiovascular risk. *Cochrane Database Syst Rev* **12**: CD002128.
116. Rees K, Hartley L, Day C et al. (2013) Selenium supplementation for the primary prevention of cardiovascular disease. *Cochrane Database Syst Rev* **1**: CD009671.
117. Rees K, Takeda A, Martin N et al. (2019) Mediterranean‐style diet for the primary and secondary prevention of cardiovascular disease. *Cochrane Database Syst Rev* **3:** CD009825.
118. Rees K, Al-Khudairy L, Takeda A et al. (2021). Vegan dietary pattern for the primary and secondary prevention of cardiovascular diseases. *Cochrane Database Syst Rev* ***2****:* CD013501.pub2
119. Ried K, Fakler P, Stocks NP (2017). Effect of cocoa on blood pressure. *Cochrane Database Syst Rev* **4:** CD008893.
120. Robertson LM, Waugh N, Robertson A. (2007) Protein restriction for diabetic renal disease. *Cochrane Database Syst Rev* **4**: CD002181.
121. Rolim LCSP, da Silva EMK, Flumignan RLG et al. (2019) Acetyl-l-carnitine for the treatment of diabetic peripheral neuropathy. *Cochrane Database Syst Rev* **6**: CD011265*.*
122. Sastre AA, Vernooij RW, Harmand MG-C et al. (2017) Effect of the treatment of Type 2 diabetes mellitus on the development of cognitive impairment and dementia. *Cochrane Database Syst Rev* **6**: CD003804.
123. Schandelmaier S, Briel M, Saccilotto R et al. (2017) Niacin for primary and secondary prevention of cardiovascular events. *Cochrane Database Syst Rev* ***6****:* CD009744.
124. Semlitsch T, Jeitler K, Berghold A et al. (2016) Long‐term effects of weight‐reducing diets in people with hypertension. *Cochrane Database Syst Rev* **3**: CD008274.
125. Shaikh H, Bradhurst P, Ma LX et al. (2020) Body weight management in overweight and obese breast cancer survivors. *Cochrane Database Syst Rev* **12**: CD012110.
126. Smart NA, Marshall BJ, Daley M et al. (2011) Low‐fat diets for acquired hypercholesterolaemia. Cochrane Database Syst Rev **2**: CD007957.pub2/full
127. Sowerbutts AM, Lal S, Clamp A et al. (2017) Home parenteral nutrition for people with inoperable malignant bowel obstruction. *Cochrane Database of Systematic Reviews* **8:** CD012812.
128. Stabler SN, Tejani AM, Huynh F et al. (2012) Garlic for the prevention of cardiovascular morbidity and mortality in hypertensive patients. *Cochrane Database Syst Rev* **8**: CD007653.
129. Suckling RJ, He FJ, MacGregor GA. (2010) Altered dietary salt intake for preventing and treating diabetic kidney disease. *Cochrane Database Syst Rev* 12: CD006763.
130. Tasnim S, Tang C, Musini VM et al. (2020). Effect of alcohol on blood pressure. Cochrane Database of Systematic Reviews **7**: CD012787.
131. Thien FC, Luca SD, Woods RK et al. (2000) Dietary marine fatty acids (fish oil) for asthma in adults and children. *Cochrane Database Syst Rev* **4**: CD001283.
132. .Thomas D, Elliott EJ (2009). Low glycaemic index, or low glycaemic load, diets for diabetes mellitus. *Cochrane Database Syst Rev* **1**: CD006296.
133. Thomas D, Elliott EJ, Baur L (2007). Low glycaemic index or low glycaemic load diets for overweight and obesity. *Cochrane Database Syst Rev* **3:** CD005105.
134. Thompson RL, Summerbell CD, Hooper L et al. (2003) Dietary advice given by a dietitian versus other health professional or self‐help resources to reduce blood cholesterol. *Cochrane Database Syst Rev* **3**: CD001366.
135. Tian H, Guo X, Wang X, He Z et al. (2013) Chromium picolinate supplementation for overweight or obese adults. Cochrane Database Syst Rev **11**: CD010063.
136. Usinger L, Reimer C, Ibsen H (2012). Fermented milk for hypertension. *Cochrane Database Syst Rev* **4**: CD008118.
137. Uthman OA, Hartley L, Rees K et al. (2015). Multiple risk factor interventions for primary prevention of cardiovascular disease in low-and middle-income countries. *Cochrane Database Syst Rev* **8**: CD011163.
138. Vinceti M, Filippini T, Giovane CD et al. (2018) Selenium for preventing cancer. *Cochrane Database Syst Rev* **1**: CD005195.
139. Von Philipsborn P, Stratil JM, Burns J et al. (2019) Environmental interventions to reduce the consumption of sugar-sweetened beverages and their effects on health. *Cochrane Database Syst Rev* ***6****:* CD012292*.*
140. Ward EJ, Henry LM, Friend AJ et al. (2015) Nutritional support in children and young people with cancer undergoing chemotherapy. *Cochrane Database Syst Rev* **8**: CD003298.
141. Wei D, Heus P, van de Wetering FT et al. (2018) Probiotics for the prevention or treatment of chemotherapy- or radiotherapy-related diarrhoea in people with cancer. *Cochrane Database Syst Rev* **8**: CD008831*.*
142. Weingarten MAM, Trestioreanu AZ, Yaphe J (2008) Dietary calcium supplementation for preventing colorectal cancer and adenomatous polyps. *Cochrane Database Syst Rev* **1**: CD003548.
143. Welsh EJ, Bara A, Barley E et al. (2010) Caffeine for asthma. *Cochrane Database Syst Rev* **1**: CD001112.
144. Wieland LS, Falzon L, Sciamanna CN et al. (2012) Interactive computer‐based interventions for weight loss or weight maintenance in overweight or obese people. *Cochrane Database Syst Rev* **8**: CD007675.
145. Wilkinson EA. (2014) Oral zinc for arterial and venous leg ulcers. *Cochrane Database Syst Rev* **9**: CD001273.
146. Wilkinson M, Hart A, Milan SJ et al. (2014) Vitamins C and E for asthma and exercise‐induced bronchoconstriction. *Cochrane Database Syst Rev* **6**: CD010749.
147. Wolfenden L, Nathan NK, Sutherland R. (2017) Strategies for enhancing the implementation of school-based policies or practices targeting risk factors for chronic disease. *Cochrane Database Syst Rev* **11:** CD011677.
148. Wolfenden L, Barnes C, Jones J et al. (2020) Strategies to improve the implementation of healthy eating, physical activity and obesity prevention policies, practices or programmes within childcare services. *Cochrane Database Syst Rev* ***2:*** CD011779.
149. Yao Y, Suo T, Andersson R, Cao Y et al. (2017) Dietary fibre for the prevention of recurrent colorectal adenomas and carcinomas. *Cochrane Database Syst Rev* **1**: CD003430.
150. Zhou Y, Yang M, Dong BR. (2012) Monosodium glutamate avoidance for chronic asthma in adults and children. Cochrane Database Syst Rev 6: CD004357.
